# Diagnostic Performance of Machine Learning for Systemic Lupus Erythematosus: Systematic Review and Meta-Analysis

**DOI:** 10.2196/90209

**Published:** 2026-09-04

**Authors:** Bingduo Wang, Zichao Wang, Yang Liu, Fangfang Ge

**Affiliations:** 1 Department of Anesthesiology and Intensive Care, The First Affiliated Hospital, Zhejiang University School of Medicine, Hangzhou, China; 2 Department of Epidemiology and Statistics, Institute of Basic Medical Sciences Chinese Academy of Medical Sciences, School of Basic Medicine Peking Union Medical College, Beijing, China; 3 Department of Proctology, The First Affiliated Hospital of Xinjiang Medical University, Xinjiang, China; 4 Department of Hematology, The First Affiliated Hospital of Zhengzhou University, No. 1, Jianshe East Road, Erqi District, Zhengzhou, China, 86 15067607819

**Keywords:** machine learning, systemic lupus erythematosus, diagnostic test accuracy, systematic review, meta-analysis, artificial intelligence

## Abstract

**Background:**

Early and accurate diagnosis of systemic lupus erythematosus (SLE) and its organ involvement is essential. Previous reviews of machine learning (ML) in SLE combined heterogeneous tasks and validation strategies and may have overinterpreted model performance.

**Objective:**

This study evaluated the diagnostic performance of ML and deep learning (DL) models for 3 clinically distinct SLE-related tasks: SLE classification or diagnosis, lupus nephritis (LN) diagnosis, and neuropsychiatric systemic lupus erythematosus (NPSLE) discrimination. We also assessed methodological quality and certainty of evidence.

**Methods:**

PubMed, Embase, Cochrane Library, Web of Science, and IEEE Xplore were searched from January 2014 to April 2026. Eligible peer-reviewed diagnostic accuracy studies developed or validated ML or DL models for 1 of the 3 prespecified tasks, used an accepted reference standard, and provided data for a 2×2 contingency table. Bivariate random-effects meta-analyses with the Hartung-Knapp-Sidik-Jonkman adjustment were used to pool sensitivity and specificity. We reported 95% prediction intervals (PIs), assessed risk of bias using the Quality Assessment of Diagnostic Accuracy Studies for Artificial Intelligence tool (QUADAS-AI; Viknesh Sounderajah [Imperial College London]), and evaluated certainty of evidence using the Grading of Recommendations Assessment, Development, and Evaluation framework for diagnostic test accuracy.

**Results:**

Twenty-nine studies were included: 17 for SLE classification, 5 for LN diagnosis, and 7 for NPSLE discrimination. In the primary task-stratified analysis, pooled sensitivity was 0.91 (95% CI 0.86-0.94; 95% PI 0.56-0.99), and pooled specificity was 0.94 (95% CI 0.91-0.96; 95% PI 0.69-0.99), with low heterogeneity (*I*²=23.9% and 22.9%, respectively). DL models showed a sensitivity of 0.93 and specificity of 0.95, compared with 0.88 and 0.94 for traditional ML models. Certainty of evidence was high for most analyses but low for LN diagnosis because of inconsistency and imprecision. All studies were retrospective, and only 9 of 29 (31%) performed independent external validation. Overall risk of bias was high or unclear in 22 of 29 (75.9%) studies. No study reported model calibration, decision-curve analysis, or net clinical benefit.

**Conclusions:**

ML models showed promising diagnostic accuracy across 3 distinct SLE-related tasks, but wide PIs, limited external validation, and pervasive risk of bias restrict conclusions about real-world generalizability. Prospective multicenter studies with standardized tasks and reference standards, independent external validation, and formal assessment of calibration and clinical utility are required before clinical implementation.

## Introduction

Systemic lupus erythematosus (SLE) is an autoimmune disease that significantly affects multiple systems and organs [1,2]. It is clinically manifested as dermatitis, lupus nephritis (LN), polyarthritis, pericarditis, and neuropsychiatric dysfunction [1,3,4]. These manifestations during disease progression complicate the accurate diagnosis [5]. SLE is characterized by immune complexes and the hyperreactivity of B cells and T cells, leading to loss of immune tolerance for circulating antibodies in affected patients [6,7]. Additionally, typical autoantibodies in the serum are elevated 3 to 9 years before clinical symptoms appear, particularly anti–double-stranded DNA, anti-Smith, and antiphospholipid antibodies [8-10]. Hence, these biomarkers have been listed in the latest 2023 European League Against Rheumatism (EULAR) and the American College of Rheumatology (ACR) criteria for SLE classification to guide thorough assessment to mitigate the risk of misdiagnosis [11].

Despite the existing knowledge, a recent study conducted by Adamichou et al [8] suggested that up to 20% of early cohort patients could not be diagnosed using the 2019 EULAR or ACR criteria, and the Systemic Lupus Collaborating Clinics (SLICC)-2012 criteria. However, the integration of EULAR or ACR and the SLICC criteria through machine learning (ML) considerably enhanced sensitivity (from 80% to 97%) for early cases [12]. Notably, neuropsychiatric symptoms of lupus are frequently observed among pediatric patients [13,14]. However, clinical examinations of cerebrospinal fluid and serum and electroencephalograms (EEGs) all fail to diagnose early-onset SLE [15]. Currently, it remains unclear whether a comprehensive analysis of all features can aid in the early diagnosis and timely treatment of these patients [16]. ML can identify patterns in large, complex datasets and support diagnostic decision-making [17].

Over the past 5 decades, since the initial discussions on AI in the medical field, ML and deep learning (DL) have made significant strides, enabling them to effectively learn from accumulated knowledge and statistical data [18,19,20]. In addition to the advantages of automation and efficiency, AI-based algorithms enhance scalability and improve decision-making processes for health care systems [21]. Recently, electronic medical records have been widely adopted, and biogenetic factors have become more intricate, which has made the need for advanced technological solutions more urgent than ever [9,22]. AI is emerging as the most suitable option to address this challenge [23]. Interestingly, Wu et al [14] developed a diagnostic model to classify metastasis stages of bladder cancer, with nearly 100% sensitivity. Therefore, given the high expectation for AI, its application in disease detection and classification is imperative, as it has the potential to revolutionize conventional clinical diagnostic approaches [24].

To ground this work in the broader context of AI in medical diagnostics, we build on recent advances in the field: AI-driven medical image analysis has demonstrated robust diagnostic performance across a wide range of clinical indications, with DL models proving particularly effective at extracting complex patterns from multimodal medical data [25,26]. DL has also been validated for standardized assessment of autoimmune disease-related imaging, reducing interreader variability and improving diagnostic efficiency in rheumatology [27]. These advances provide a strong foundational rationale for exploring ML for SLE diagnosis, but also highlight the need for rigorous, clinically oriented, and comprehensive analysis of the evidence to guide clinical translation [28-30].

Despite the rapid growth of research in this field, existing systematic reviews on the application of ML for SLE diagnosis have critical, fundamental limitations that undermine the validity of their conclusions [31]. First, and most importantly, prior syntheses have pooled heterogeneous, clinically incomparable tasks—including SLE classification, LN activity grading, neuropsychiatric systemic lupus erythematosus (NPSLE) vs multiple sclerosis discrimination, and disease activity score estimation—into a single meta-analysis, which directly violates the core assumptions of diagnostic test accuracy (DTA) meta-analysis and results in pooled estimates that lack clinical interpretability [32,33]. Second, previous reviews have conflated results from internal validation (which is well known to systematically overestimate model performance) and independent external validation, leading to an overestimation of real-world generalizability [34]. Third, no prior review has used state-of-the-art statistical methods for DTA meta-analysis, including the Hartung-Knapp-Sidik-Jonkman (HKSJ) random-effects model and prediction intervals (PIs); these methods are essential for addressing extreme between-study heterogeneity and quantifying real-world performance variability [35]. Fourth, existing syntheses have not yet used the QUADAS-AI (Quality Assessment of Diagnostic Accuracy Studies for Artificial Intelligence) tool (Viknesh Sounderajah [Imperial College London]), the validated gold standard for AI-based diagnostic studies, to comprehensively assess the methodological quality of included studies, nor have they addressed the widespread risk of optimism bias from selective reporting of best-performing models [36]. Finally, no review has systematically evaluated the critical gap in clinical utility assessment, including model calibration, net clinical benefit, and workflow integration, which are essential to determine whether ML models will improve patient outcomes in real-world clinical settings [37].

This systematic review and meta-analysis addresses all of these critical gaps in existing literature, with 4 core innovations and contributions to the field. First, with respect to methodological rigor, we perform the first fully task-stratified DTA meta-analysis, with primary analyses restricted to 3 independent, clinically homogeneous diagnostic tasks (SLE classification, LN diagnosis, and NPSLE discrimination), eliminating the core flaw of heterogeneous task pooling that invalidated prior reviews. Second, with respect to statistical methodology, we use the HKSJ random-effects model (recommended for DTA meta-analysis) and 95% PIs to quantify real-world performance variability, reporting PI lines in all forest plots and avoiding overinterpretation of pooled point estimates in the context of heterogeneity. Third, with respect to bias mitigation, we implement a strict, prespecified rule for contingency table inclusion, with each study contributing only one independent table to primary analyses (prioritizing external validation and prespecified model results, rather than post hoc best-performing models), eliminating data dependency, double counting, and optimism bias. Fourth, with respect to clinical relevance, we systematically distinguish internal versus external validation results, comprehensively appraise study quality with QUADAS-AI, perform GRADE (Grading of Recommendations Assessment, Development, and Evaluation) assessment of certainty of evidence, and explicitly address the absence of clinical utility assessment in existing research, providing evidence-based guidance for both future research and clinical implementation.

## Methods

### Protocol and Registration

This systematic review and meta-analysis was conducted in strict accordance with the PRISMA (Preferred Reporting Items for Systematic Reviews and Meta-Analyses) 2020 statement (Checklist 1), PRISMA-DTA (PRISMA for Diagnostic Test Accuracy) guidelines, and PRISMA-S (Preferred Reporting Items for Systematic Reviews and Meta-Analyses literature search extension) reporting standards. The study protocol was preregistered in the International Prospective Register of Systematic Reviews (PROSPERO) on May 7, 2024 (registration number: CRD42024545109). All deviations from the preregistered protocol are explicitly listed in Multimedia Appendix 1, with a rationale for each change. Ethical approval and informed consent were not required for this systematic review of published literature [15]. The completed PRISMA 2020, PRISMA-DTA, and PRISMA-S checklists are provided in Checklist 1–4. The following PRISMA-S items were not applicable to this review: (1) item 12 (automated search alerts or updates): no automated alerts were set, as the search was rerun manually at the time of revision; (2) item 13 (gray literature and other resources): the review was restricted to full-text peer-reviewed publications, and no gray literature, trial registries, or unpublished sources were searched; and (3) item 14 (contacting authors for unpublished data): authors were not contacted, as only published diagnostic accuracy data permitting construction of 2×2 contingency tables were eligible for inclusion. These decisions are consistent with the preregistered study protocol (PROSPERO CRD42024545109).

### Literature Search Strategy

A comprehensive, peer-reviewed literature search was developed in collaboration with a medical librarian, in full adherence to the Cochrane Handbook for Systematic Reviews of Diagnostic Test Accuracy and PRISMA-S guidelines. The search was originally conducted through January 2025 and was fully updated through April 2026 at the time of revision. PubMed, Embase, Cochrane Library, Web of Science, and IEEE Xplore were searched for studies published between January 1, 2014, and April 2026. No geographical restrictions were applied, and only studies published in English were included.

The search strategy combined MeSH terms, Emtree terms, and free-text keywords for three core concepts: (1) SLE and related manifestations, (2) ML and AI, and (3) DTA. Field modifiers were used to restrict terms to the title or abstract fields, where appropriate, to maximize sensitivity and specificity. The full, unedited search strategy for each database is provided in Multimedia Appendix 2.

Additional studies were identified through manual screening of the reference lists of included studies and relevant systematic reviews, to ensure that no eligible studies were missed.

### Study Selection Criteria

Studies were eligible if they met all of the following criteria. First, regarding study design, eligible studies were full-text, peer-reviewed diagnostic accuracy studies that developed or validated ML or DL models for 1 of the 3 prespecified, clinically homogeneous diagnostic tasks (SLE classification, LN diagnosis, or NPSLE discrimination). Second, regarding the index test, eligible studies used ML or DL models with any type of clinical input data, including histopathology images, radiological images (magnetic resonance imaging [MRI], computed tomography [CT], and ultrasound), spectroscopic data (Raman spectroscopy and Fourier transform infrared spectroscopy [FTIR]), and electronic health record (EHR) data. Third, regarding the reference standard, a clinically accepted independent reference standard was required: for SLE classification, the 1997 ACR, 2012 SLICC, or 2019 EULAR or ACR criteria, or expert consensus by board-certified rheumatologists; for LN diagnosis, renal biopsy (International Society of Nephrology [ISN] or Renal Pathology Society [RPS] classification); and for NPSLE discrimination, the 1999 ACR NPSLE definitions with expert consensus. Fourth, regarding outcome data, studies were required to report sufficient information to construct a 2×2 contingency table (true positive [TP], false positive [FP], false negative [FN], and true negative [TN]) or to provide sensitivity, specificity, and sample size to allow calculation of these values.

Studies were excluded if they met any of the following criteria: nonhuman or animal studies; review articles, editorials, case reports, conference abstracts, letters, or study protocols; studies focused solely on disease prognosis, treatment response prediction, or disease activity score estimation without a primary diagnostic outcome; studies that did not use an independent reference standard or where the reference standard was partially derived from the ML model input features (circular validation); duplicate publications of the same study cohort; or studies with a sample size of fewer than 20 participants.

Two independent reviewers (BW and ZW) performed title and abstract screening in duplicate, followed by full-text screening of potentially eligible studies. Disagreements were resolved by consensus with a third senior reviewer (FG).

### Data Extraction

Data extraction was performed independently by 3 reviewers (BW, ZW, and YL) using a prepiloted, standardized data extraction form. The form was expanded to include items specific to AI-based diagnostic studies, and all extracted data were cross-checked for accuracy. Discrepancies were resolved by consensus with a fourth reviewer (FG).

The following data were extracted from each included study. Study characteristics included first author, year of publication, study country, study design, study period, clinical setting, sample size, participant demographics, and prespecified inclusion and exclusion criteria. Clinical task details included the explicit definition of the diagnostic task, target population, and clinical decision context. Index test details included the type of ML or DL algorithm, input data modality, model preprocessing steps, training, validation, and testing workflow, and whether the reported model was prespecified or post hoc selected as the best-performing model. Validation strategy details included clear definitions of training, validation, and test sets; whether patient-level splits were used; whether internal or independent external validation was performed; and whether data leakage was assessed and excluded. Reference standard details included the type of reference standard used, definition of positive and negative cases, and whether the reference standard assessment was blinded to the index test results. Diagnostic accuracy outcomes included TP, FP, FN, TN, sensitivity, specificity, area under the curve (AUC), positive predictive value (PPV), negative predictive value (NPV), and 95% CIs, with separate extraction for internal and external validation cohorts. Secondary outcomes included head-to-head comparisons with human clinicians, model calibration, decision-curve analysis (DCA), net clinical benefit, and implementation outcomes. Risk of bias items included all items required for the QUADAS-AI risk of bias assessment.

### Rules for Including Contingency Tables (Prespecified to Eliminate Bias and Data Dependency)

To avoid double-counting, data dependency, and optimism bias, we implemented the following strict, prespecified rules for including contingency tables in meta-analyses. The following prespecified rules governed contingency table inclusion. For the primary meta-analyses, each independent study cohort contributed exactly one 2×2 contingency table, selected according to the following priority order: first, results from an independent external validation cohort for a prespecified model; second, results from an internal validation cohort for a prespecified model; third, results from the full study cohort for a prespecified model. Post hoc selected best-performing model results were explicitly excluded from all primary analyses to eliminate researcher-driven optimism bias. For subgroup and sensitivity analyses, multiple nonoverlapping contingency tables from the same study were included only if they derived from mutually exclusive patient cohorts with no overlapping participants; all nonindependent data were explicitly labeled and their limitations acknowledged in the paper. Two levels of exploratory analyses were conducted: (1) an exploratory all-task pooled analysis using one table per study (selected using the priority order above) to provide an overall summary, with a clear statement that this analysis has no clinical interpretability; and (2) an exploratory all-table analysis in which all eligible algorithm-level contingency tables from each study were included (124 tables from 29 studies [8,16,17,19-32,34-45]), explicitly treated as exploratory because multiple tables could originate from the same study, potentially introducing within-study correlation.

### Risk of Bias Assessment

The risk of bias and applicability concerns of each included study were assessed independently by 2 reviewers (BW and FG) using the QUADAS-AI tool, the validated gold standard for AI-based diagnostic accuracy studies. The QUADAS-AI tool comprises 4 domains: patient selection, index test, reference standard, and flow and timing. Each domain was rated for risk of bias (low, high, or unclear) and applicability concerns (low, high, or unclear), with explicit justifications for each rating. Disagreements were resolved by consensus with a third reviewer (YL).

### Statistical Analysis

All statistical analyses were prespecified in the study protocol and performed using R (version 4.4.2; R Core Team) with the mada package for bivariate random-effects meta-analyses. Statistical significance was assessed using a 2-sided α level of .05.

### Primary Meta-Analyses

For each of the 3 independent clinical tasks, we performed hierarchical summary receiver operating characteristic (HSROC) meta-analyses to estimate pooled sensitivity, specificity, and AUC. The HKSJ random-effects model was used for all pooled analyses, as this method provides more robust type I error control and more precise effect estimates than the standard DerSimonian-Laird method, particularly in the setting of high between-study heterogeneity and small numbers of included studies.

For all pooled sensitivity and specificity estimates, we reported the pooled point estimate, 95% CI, which quantifies uncertainty around the average effect, and 95% PI, which quantifies the expected range of true effect sizes across different settings for 95% of future similar studies, and provides a clinically relevant estimate of real-world performance variability. The CI and PI serve fundamentally different purposes. The CI reflects the precision of the pooled mean, whereas the PI reflects the distribution of expected effects in new settings, accounting for between-study variance [35].

Between-study heterogeneity was quantified using the *I*² statistic, with the following thresholds: *I*²<50%= low heterogeneity, 50%‐75%=moderate heterogeneity,>75%= high heterogeneity,>90%= extreme heterogeneity. The *I*² statistic has limited utility in practical applications because it cannot quantify the magnitude of true effect variation across populations. Therefore, the 95% PI is used as the primary measure of real-world heterogeneity. For analyses with extreme heterogeneity (*I*²>90%), the pooled point estimate has limited clinical significance, and the PI should be used to interpret real-world performance.

### GRADE Certainty-of-Evidence Assessment

The certainty of evidence for each analysis was evaluated using the GRADE framework adapted for DTA studies [46,47]. The certainty started at high and was rated down for five domains: (1) risk of bias, downgraded if ≥1/3 of studies had any QUADAS-AI domain rated as high risk; (2) inconsistency, downgraded one level if *I*²>50% and 2 levels if *I*²>75% for either sensitivity or specificity; (3) indirectness, downgraded if the study populations, index tests, or reference standards did not directly match the review question; (4) imprecision, downgraded if the number of studies was<5 or the maximum 95% CI width for pooled sensitivity or specificity exceeded 0.20; and (5) publication bias, downgraded if Deeks asymmetry test met the prespecified significance threshold of α=.10. The GRADE assessment was performed for the overall analysis, ML and DL subgroups, each clinical task, and the external validation subgroup.

### Subgroup and Sensitivity Analyses

Prespecified subgroup analyses were performed to explore potential sources of heterogeneity, using the HKSJ model, across the following dimensions: validation strategy (internal validation vs external validation); algorithm type (traditional ML vs DL); input data modality (histopathology vs radiological imaging vs spectroscopic data vs EHR data); sample size (≥100 participants vs <100 participants); and risk of bias (low overall risk vs high or unclear overall risk).

Prespecified sensitivity analyses were performed using the leave-one-out analysis (sequentially removing each study to assess its impact on the pooled estimate), by excluding studies with a high overall risk of bias, and by excluding studies with a sample size<50 participants to assess the robustness of the primary pooled estimates.

### Small-Study Effects Assessment

Visual inspection of funnel plots and the Deeks funnel plot asymmetry test were used to assess small-study effects. These methods can only assess small-study effects, not publication bias, as publication bias is only one of the many potential causes of small-study effects. Additionally, these analyses have limited statistical power with a small number of included studies and cannot rule out researcher-driven optimism bias from selective reporting of favorable results.

### Comparison Between ML Models and Human Clinicians

Given that only 2 studies [17,40] reported head-to-head comparisons between ML models and human clinicians, with sparse data, heterogeneous clinical tasks, and varying clinician expertise levels, we performed an exploratory descriptive summary receiver operating characteristic (SROC) analysis only to visualize the comparative diagnostic space; no inferential meta-analysis or formal between-group statistical comparison was attempted, as the data were insufficient to produce reliable pooled estimates.

### Ethical Considerations

As this is a systematic review and meta-analysis of published literature, ethical approval and informed consent are not applicable.

## Results

### Study Selection

**Figure 1.**
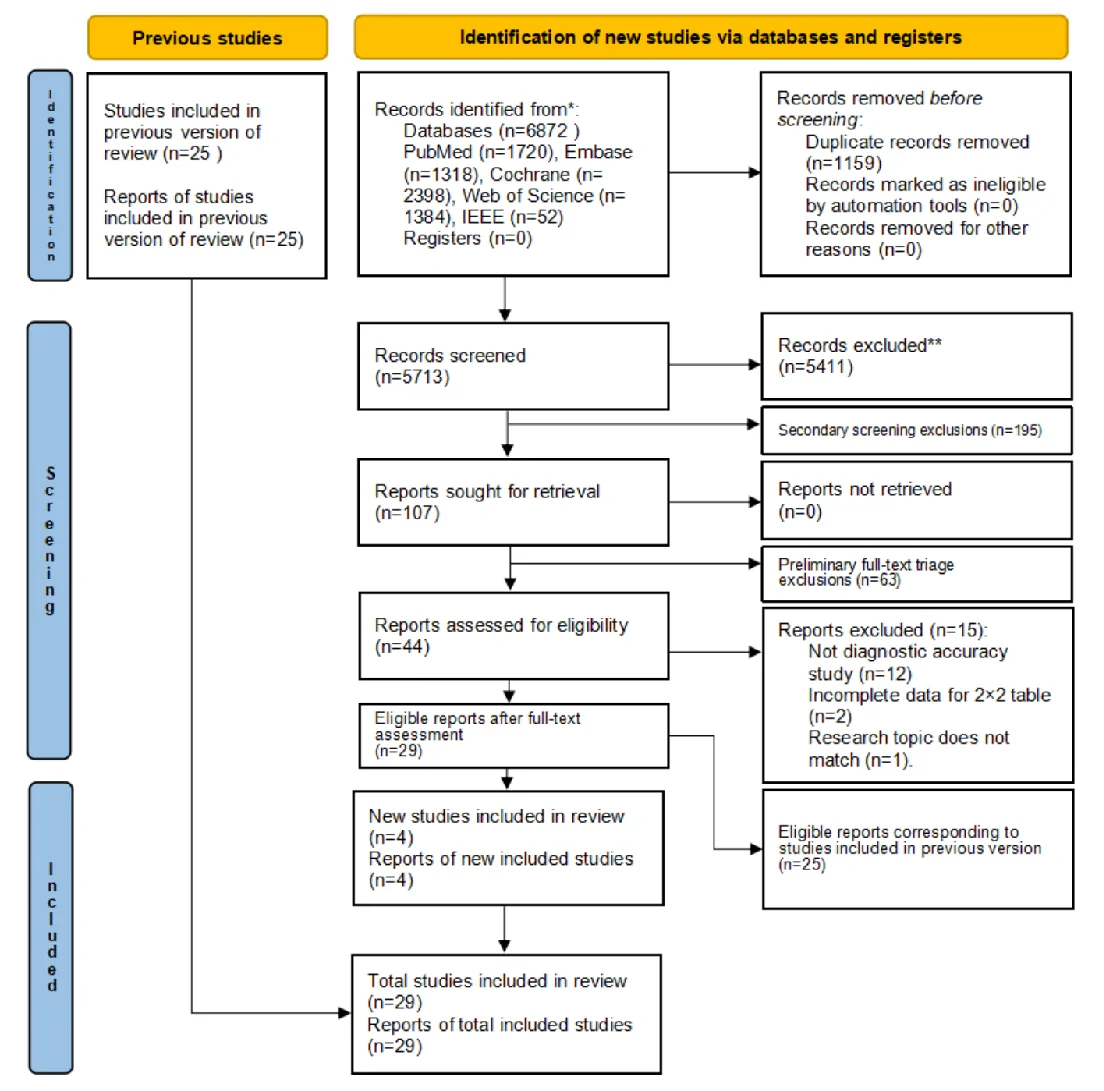
PRISMA (Preferred Reporting Items for Systematic Reviews and Meta-Analyses) 2020 flow diagram of study selection.

A total of 6872 records were retrieved from the electronic database search, with 1159 duplicate records removed, leaving 5713 records for screening. Of these, 5411 records were excluded during initial title and abstract screening, leaving 302 potentially relevant records. A further 195 records were excluded during secondary eligibility screening before full-text retrieval, leaving 107 reports sought for retrieval. All 107 reports were retrieved. Sixty-three reports were excluded during preliminary full-text triage before formal eligibility assessment, leaving 44 reports assessed in detail. Of these, 15 were excluded for the following reasons: not a diagnostic accuracy study (n=12), incomplete data for construction of a 2×2 table (n=2), and research topic mismatch (n=1). The remaining 29 eligible studies comprised 25 studies included in the previous version of the review [8,16,17,19-32,34-41] and 4 newly included studies [42-45], yielding a total of 29 studies in the updated review. The detailed study selection process is shown in Figure 1.

**Figure 2.**
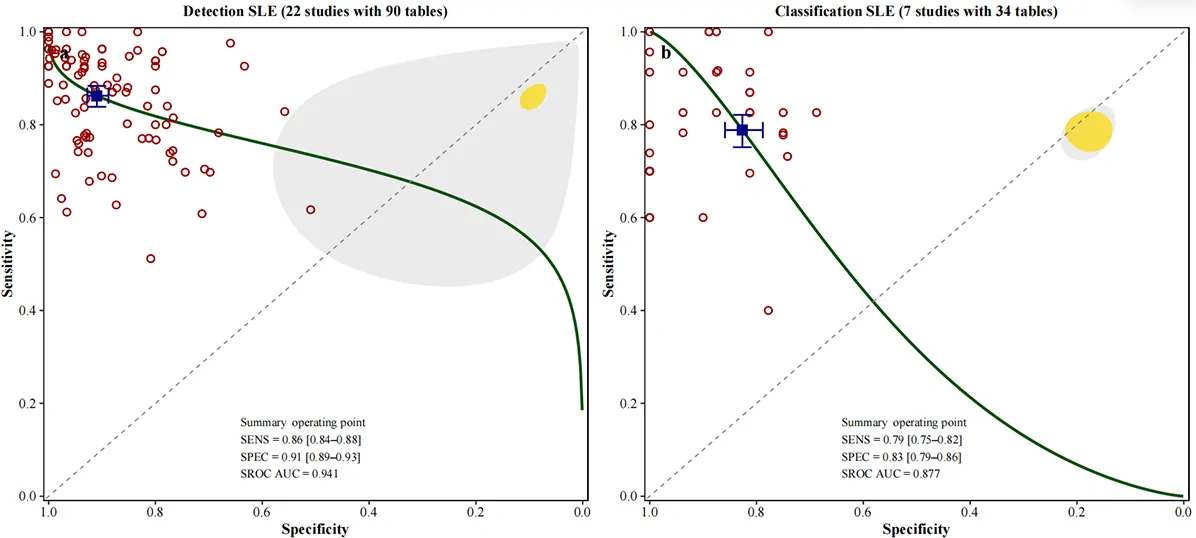
Pooled performance stratified by task type (exploratory analysis). (A) Receiver operating characteristic curves for systemic lupus erythematosus detection (22 studies with 90 tables) [8,19-24,26,27,29-32,34-37,40-44] and (B) receiver operating characteristic curves for systemic lupus erythematosus classification (7 studies with 34 tables) [16,17,25,28,38,39,45]. AUC: area under the curve; SENS: sensitivity; SLE: systemic lupus erythematosus; SPEC: specificity; SROC: summary receiver operating characteristic.

**Figure 3.**
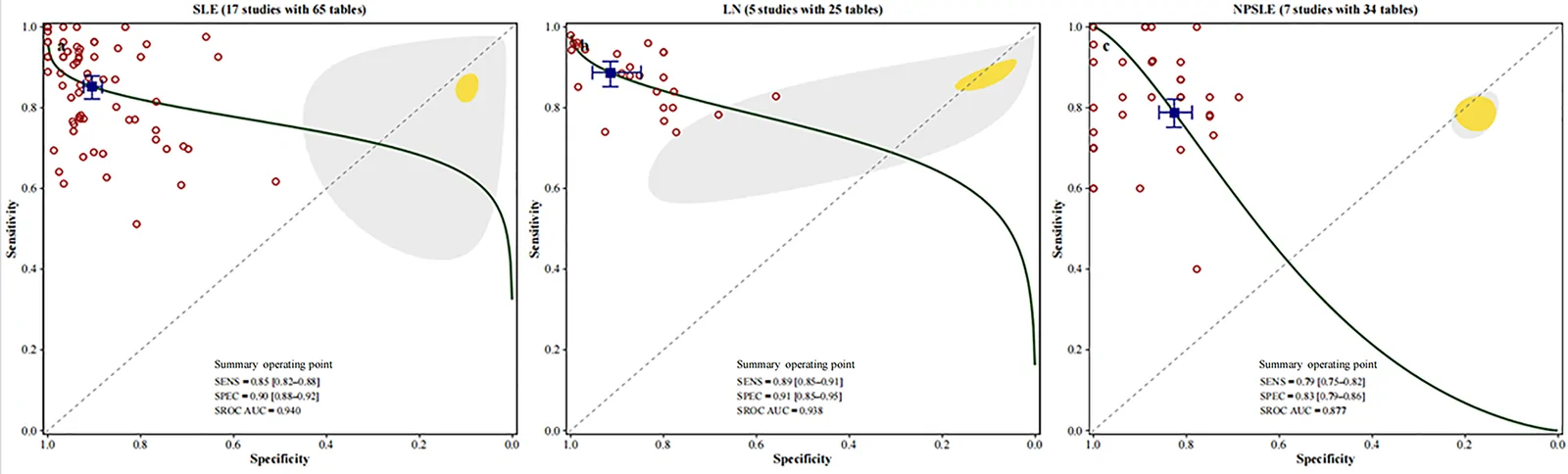
Pooled performance stratified by disease type (exploratory analysis). (A) Systemic lupus erythematosus (17 studies with 65 tables) [8,21,23,24,27,29-31,34-37,40-44], (B) lupus nephritis (5 studies with 25 tables) [19,20,22,26,32], and (C) neuropsychiatric systemic lupus erythematosus (7 studies with 34 tables) [16,17,25,28,38,39,45]. AUC: area under the curve; LN: lupus nephritis; NPSLE: neuropsychiatric systemic lupus erythematosus; SENS: sensitivity; SLE: systemic lupus erythematosus; SPEC: specificity; SROC: summary receiver operating characteristic.

A total of 29 studies met all criteria for the quantitative meta-analysis [8,16,17,19-32,34-45]. Aliyev et al [48] and de Araújo et al [33] were excluded from all analyses. The 29 studies were distributed across the 3 prespecified clinical tasks as follows: 17 studies for SLE classification [8,21,23,24,27,29-31,34-37,40-44], 5 studies for LN diagnosis [19,20,22,26,32], and 7 studies for NPSLE discrimination [16,17,25,28,38,39,45], with no overlapping patient cohorts across tasks. In the all-table exploratory analysis, 124 tables from 29 studies were included [8,16,17,19-32,34-45]. The exploratory receiver operating characteristic (ROC) results stratified by task type and disease type are shown in Figures 2 and 3, respectively. The pooled overall ROC analysis across all included studies is presented in Figure S1 in Multimedia Appendix 3. The forest plot for the all-table exploratory analysis is presented in Figure S3 in Multimedia Appendix 3.

### Study Characteristics

The baseline characteristics of the 29 included studies [8,16,17,19-32,34-45] are summarized in Tables S1-S3 in Multimedia Appendix 3. The studies were published between 2018 and 2026, with sample sizes ranging from 26 to 6476 participants. All included studies were retrospective in design. Of the 29 studies [8,16,17,19-32,34-45], 9 (31%) [21,23,24,28,32,38,40,42,45] performed independent external validation using out-of-sample, multicenter, or temporally separated cohorts; the remaining 20 (69%) [8,16,17,19,20,22,25-27,29-31,34-37,39,41,43,44] used only internal validation (random split-sample or k-fold cross-validation within a single dataset). Study design and basic demographic characteristics are summarized in Table S3 in Multimedia Appendix 3; the methods of model training and validation are summarized in Table S1 in Multimedia Appendix 3; indicators, algorithms, and data sources are summarized in Table S2 in Multimedia Appendix 3; terminology mapping across included studies is provided in Table S4 in Multimedia Appendix 3; and the implementation and reporting checklist is provided in Table S5 in Multimedia Appendix 3.

The included studies covered 3 primary clinical tasks, including SLE classification or diagnosis (17 studies) [8,21,23,24,27,29-31,34-37,40-45], LN diagnosis and classification (5 studies) [19,20,22,26,32], and NPSLE discrimination (7 studies) [16,17,25,28,38,39,45]. Input data modalities included histopathology images (9 studies, comprising 7 independent patient cohorts) [8,20-24,29,32,37], MRI (8 studies) [16,17,25,28,36,38,39,45], Raman spectroscopy (3 studies) [34,35,41], clinical images (3 studies) [27,30,40], retinal fundus imaging (1 study) [42], nailfold videocapillaroscopy (1 study) [44], optical coherence tomography (OCT; 1 study) [31], ultrasound (1 study) [26], finger optical diffusion imaging (FODI; 1 study) [19], and EHR and laboratory data (1 study) [43]. For subgroup meta-analysis by modality, retinal fundus imaging and nailfold videocapillaroscopy were grouped with clinical images (5 studies total) [27,30,40,42,44], and only modality subgroups represented by at least 3 studies were analyzed separately. Only 2 studies (6.9%) [40,42] performed head-to-head comparisons between ML models and board-certified rheumatologists or dermatologists using the same held-out test dataset. None of the included studies reported model calibration, DCA, or net clinical benefit.

### Risk of Bias Assessment

**Figure 4.**
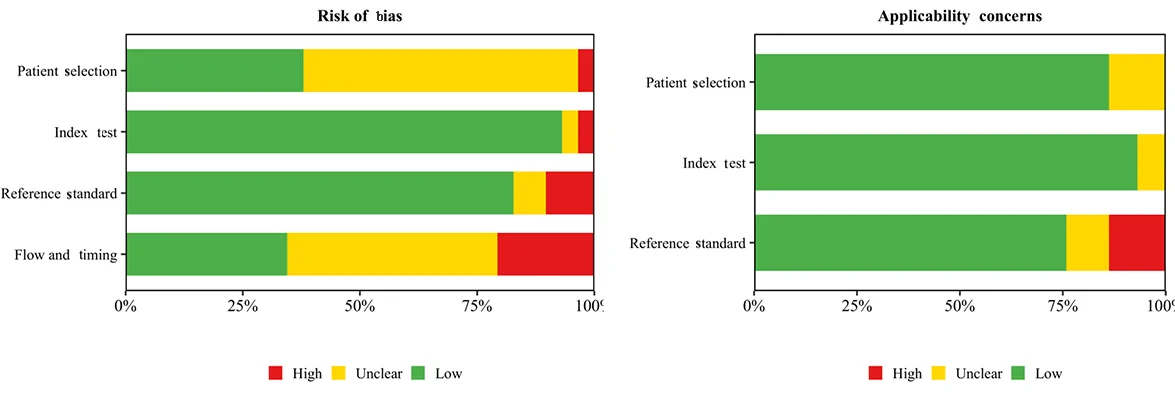
QUADAS-AI (Quality Assessment of Diagnostic Accuracy Studies for Artificial Intelligence) summary risk of bias and applicability concerns.

**Figure 5.**
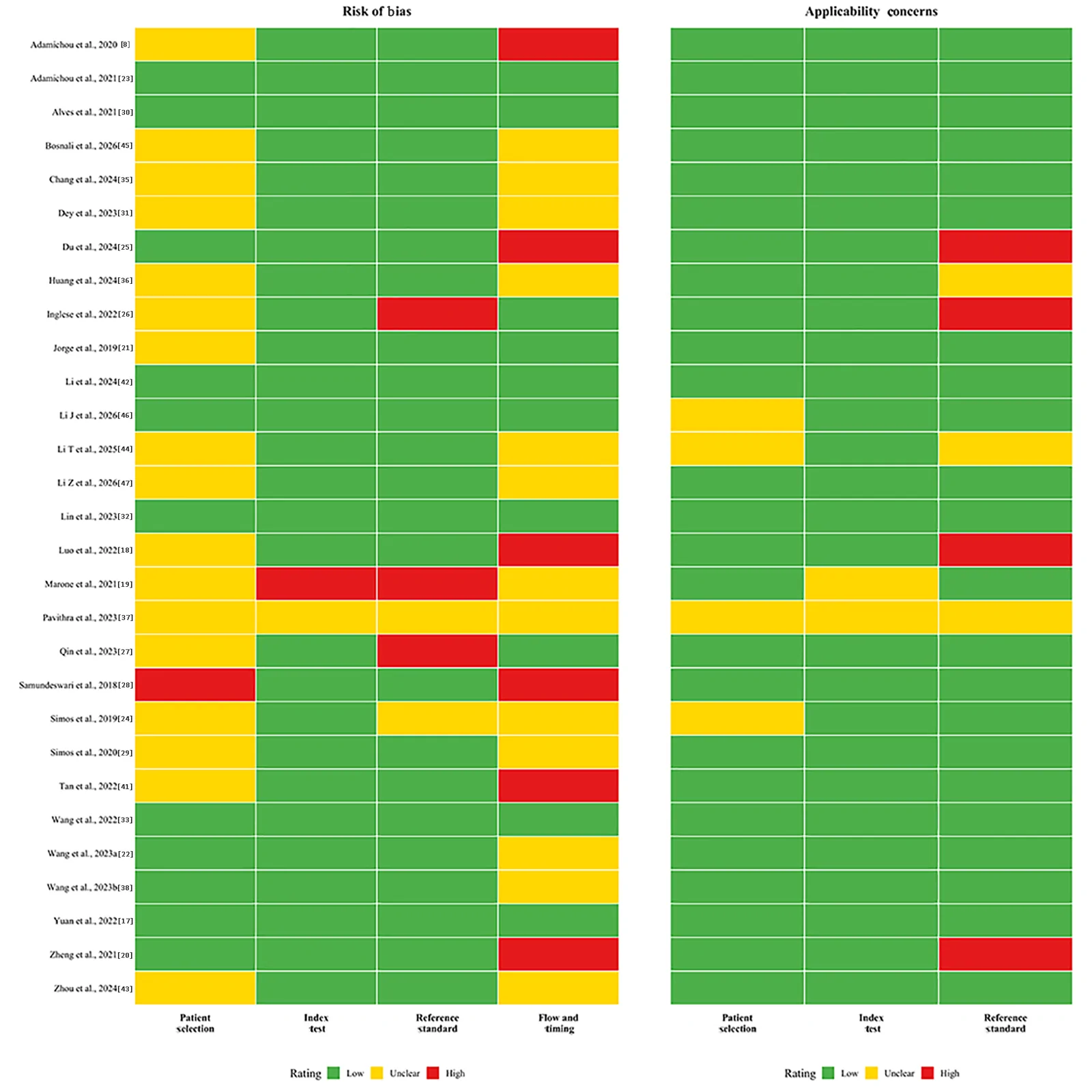
QUADAS-AI (Quality Assessment of Diagnostic Accuracy Studies for Artificial Intelligence) per-study traffic-light plot for risk of bias and applicability concerns [8,16,17,19-32,34-45].

The results of the QUADAS-AI risk of bias assessment are presented in Figures 4 and 5. Overall, 22 out of 29 studies (75.9%) [8,17,19-22,24-28,30,34-39,41-43,45] were judged to have high or unclear overall risk of bias.

In the domain of patient selection, 18 (62.1%) [8,17,19,21,25-28,30,34-36,38,39,41-43,45] studies had high or unclear risk of bias, primarily due to nonconsecutive patient recruitment, case-control design that did not reflect the clinical target population, or inappropriate exclusion criteria. For the index test, 27 (93.1%) [8,16,17,20-32,34,35,37-45] studies had low risk of bias, while 1 (3.4%) [36] study had unclear risk of bias and 1 (3.4%) study [19] had high risk of bias. For reference standards, 24 (82.8%) [8,16,17,20-24,27-32,34,35,37,39-45] studies had low risk of bias, 2 (6.9%) [36,38] studies had unclear risk of bias, and 3 (10.3%) [19,25,26] studies had high risk of bias, primarily due to lack of blinding of the reference standard assessment to the index test results or inconsistent application of the reference standard. Regarding flow and timing, 19 (65.5%) [8,17,19,20,22,24,27,28,30,34-39,41-43,45] studies had high or unclear risk of bias, primarily due to lack of reporting of the time interval between the index test and reference standard, or exclusion of participants from the final analysis without justification.

For applicability concerns, 4 (13.8%) [36,38,42,44] studies had unclear concerns related to patient selection. For the index test, 2 (6.9%) [19,36] studies had unclear concerns. For the reference standard, 3 (10.3%) [35,36,42] studies had unclear concerns, and 4 (13.8%) [17,20,24,25] studies had high concerns.

### Meta-Analyses of Task-Stratified Diagnostic Performance

**Figure 6.**
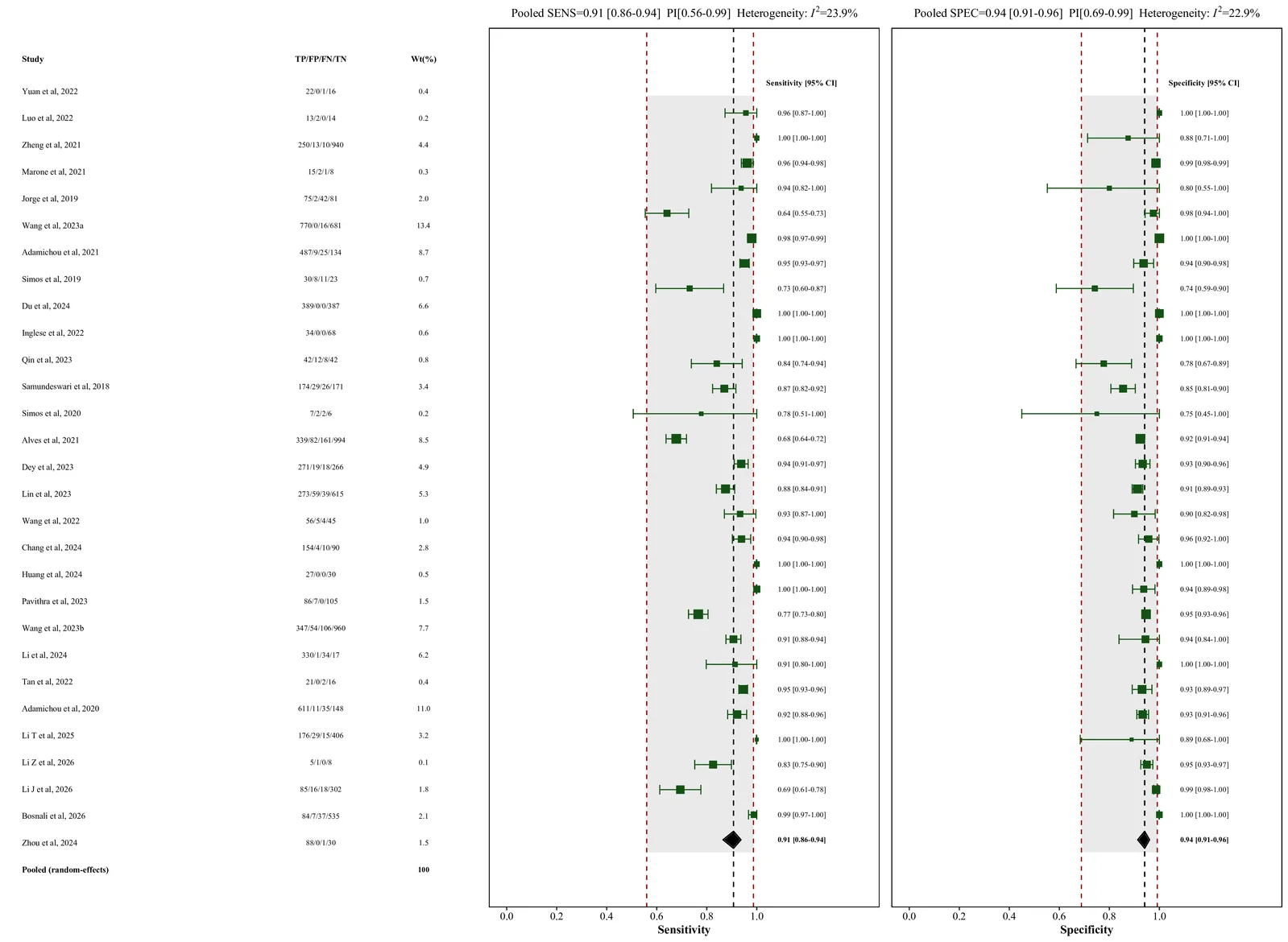
Cochrane-style forest plot for the primary diagnostic meta-analysis (29 studies) [8,16,17,19-32,34-45]. FN: false negative; FP: false positive; PI: prediction interval; SENS: sensitivity; SPEC: specificity; TN: true negative; TP: true positive; Wt: study weight. Random-effects bivariate model with Knapp-Hartung adjustment; dashed red=95% prediction interval.

The HKSJ random-effects model was used for all primary analyses, with one independent contingency table per study, prioritizing external validation and prespecified model results. In the primary analysis of all 29 studies [8,16,17,19-32,34-45], the pooled sensitivity was 0.91 (95% CI 0.86‐0.94, 95% PI 0.56‐0.99) and the pooled specificity was 0.94 (95% CI 0.91‐0.96, 95% PI 0.69‐0.99), with low between-study heterogeneity (*I*²=23.9% for sensitivity, *I*²=22.9% for specificity). Deeks funnel plot asymmetry test showed no significant small-study effects (*P*=.19). The Cochrane-style forest plot for the primary analysis is shown in Figure 6. The forest plot for the primary task-stratified analysis is also presented in Figure S2 in Multimedia Appendix 3.

A total of 17 studies (one independent contingency table per study) were included in the primary analysis for SLE classification (Task 1) [8,21,23,24,27,29-31,34-37,40-44]. The pooled results showed a sensitivity of 0.90 (95% CI 0.84‐0.94, 95% PI 0.53‐0.99) and specificity of 0.94 (95% CI 0.92‐0.96, 95% PI 0.85‐0.98). Low between-study heterogeneity (*I*²=24.7% for sensitivity, *I*²=6.7% for specificity) was observed. The CI, reflecting precision of the average effect, was relatively narrow, whereas the PI, reflecting the expected distribution of performance in new settings, was considerably wider. This distinction is critical: the narrow CI indicates that the average performance is precisely estimated, while the wide PI (sensitivity 0.53‐0.99) indicates that a new study conducted in a different setting could yield substantially different results depending on population characteristics, imaging modality, and reference standard [35]. According to the GRADE assessment, the certainty of evidence was high for SLE classification (Table 1).

**Table 1.** GRADE^a^ certainty of evidence for diagnostic test accuracy (29 studies) [8,16,17,19-32,34-45].

Outcome	Number of studies	Risk of bias	Inconsistency	Indirectness	Imprecision	Publication bias	Certainty
Overall (29 studies)	29	No serious	No serious	No serious	No serious	Undetected	High
ML^b^ subgroup	19	No serious	No serious	No serious	No serious	Not assessed	High
DL^c^ subgroup	10	No serious	No serious	No serious	No serious	Not assessed	High
SLE^d^ classification	17	No serious	No serious	No serious	No serious	Not assessed	High
LN^e^ diagnosis	5	No serious	Some inconsistency (*I*²=56%)	No serious	Imprecision (k=5, max CI width=0.23)	Not assessed	Low
NPSLE^f^ discrimination	7	No serious	No serious	No serious	No serious	Not assessed	High
External validation	9	No serious	No serious	No serious	No serious	Not assessed	High

a GRADE: Grading of Recommendations Assessment, Development and Evaluation.

b ML: machine learning.

c DL: deep learning.

d SLE: systemic lupus erythematosus.

e LN: lupus nephritis.

f NPSLE: neuropsychiatric systemic lupus erythematosus.

The GRADE framework was used to assess the certainty of evidence for DTA. Certainty starts at high and is rated down for risk of bias when at least one-third of the included studies have at least one QUADAS-AI domain rated as high risk, inconsistency (*I*²>50% one level and >75% 2 levels), indirectness, imprecision (k<5 or 95% CI width>0.20 for sensitivity or specificity), and publication bias (Deeks test significance threshold α=.10, where assessed). *I*² represents between-study heterogeneity, and CI denotes confidence interval.

Five studies (one independent contingency table per study) were included in the primary analysis for LN diagnosis (Task 2) [19,20,22,26,32]. The pooled results showed a sensitivity of 0.93 (95% CI 0.86‐0.97, 95% PI 0.68‐0.99) and specificity of 0.95 (95% CI 0.76‐0.99, 95% PI 0.19‐1.00). Low-to-moderate between-study heterogeneity (*I*²=18.4% for sensitivity, *I*²=56.4% for specificity) was observed. The wide PI for specificity (0.19‐1.00), driven by moderate heterogeneity (*I*²=56.4%), indicates variability in reference standard definitions and patient population composition across LN studies. Caution should be exercised when generalizing these estimates to new clinical settings. According to the GRADE assessment, the certainty of evidence was low for LN diagnosis, downgraded for inconsistency (*I*²=56.4% for specificity) and imprecision based on the 5 included studies [19,20,22,26,32] and a maximum CI width of 0.23 (Table 1). This low certainty of evidence indicates that the true diagnostic performance may be substantially different from the pooled estimate.

Seven studies (one independent contingency table per study) were included in the primary analysis for NPSLE discrimination (Task 3) [16,17,25,28,38,39,45]. The pooled results showed a sensitivity of 0.88 (95% CI 0.76‐0.95, 95% PI 0.54‐0.98) and specificity of 0.89 (95% CI 0.76‐0.95, 95% PI 0.50‐0.99). Between-study heterogeneity was low (*I*²=18.0% for sensitivity, *I*²=21.7% for specificity). The low heterogeneity makes the pooled point estimate more clinically interpretable. However, the wide PIs (sensitivity 0.54‐0.98, specificity 0.50‐0.99) indicate that in a new clinical setting, the expected performance could vary substantially, reflecting differences in imaging modality, patient selection criteria, and NPSLE definition across studies [49]. According to the GRADE assessment, the certainty of evidence was high for NPSLE discrimination (Table 1). The forest plots for each clinical task are presented in Figure S4 in Multimedia Appendix 3.

### Exploratory All-Task Pooled Analysis

An exploratory pooled analysis of all 29 studies (one independent table per study) [8,16,17,19-32,34-45] yielded a pooled sensitivity of 0.91 (95% CI 0.86‐0.94, 95% PI 0.56‐0.99), specificity of 0.94 (95% CI 0.91‐0.96, 95% PI 0.69‐0.99), and AUC of 0.969 (SROC), with low between-study heterogeneity (*I*²=23.9% for sensitivity, *I*²=22.9% for specificity; Deeks *P*=.19). This analysis is presented for methodological exploration only. Since these pooled point estimates combined heterogeneous and incomparable clinical tasks, they lacked clinical or statistical significance and should not be used to infer the overall diagnostic performance of ML models for SLE.

### GRADE Assessment

The GRADE assessment results are summarized in Table 1. The overall certainty of evidence was rated as high for most analyses, including the primary analysis of all 29 studies [8,16,17,19-32,34-45], the ML and DL subgroups, SLE classification, NPSLE discrimination, and external validation (Table 2). The certainty of evidence for LN diagnosis was rated as low due to inconsistency (*I*²=56.4% for specificity) and imprecision (k=5 studies [19,20,22,26,32], maximum CI width=0.23). These results indicate that while the pooled estimates for most analyses are reliable, the evidence for LN diagnosis should be interpreted with caution, and additional studies are needed to increase confidence in the pooled estimates for this task.

**Table 2.** Summary of pooled diagnostic performance across all analyses (29 studies)^a^ [8,16,17,19-32,34-45].

Analysis	Pooled sensitivity (95% CI)	95% PI^b^ (sensitivity)	Pooled specificity (95% CI)	95% PI (specificity)	*I*²^c^ sens (%)	*I*² spec (%)	Deeks *P* value
Primary (all 29 studies)	0.91 (0.86‐0.94)	0.56‐0.99	0.94 (0.91‐0.96)	0.69‐0.99	23.9	22.9	.19
ML^d^ subgroup (n=19)	0.88 (0.81‐0.93)	0.44‐0.99	0.94 (0.89‐0.97)	0.53‐0.99	27.6	32.7	—^e^
DL^f^ subgroup (n=10)	0.93 (0.91‐0.95)	0.85‐0.97	0.95 (0.93‐0.97)	0.85‐0.99	5.3	10.0	—
SLE^g^ classification (n=17)	0.90 (0.84‐0.94)	0.53‐0.99	0.94 (0.92‐0.96)	0.85‐0.98	24.7	6.7	—
LN^h^ diagnosis (n=5)	0.93 (0.86‐0.97)	0.68‐0.99	0.95 (0.76‐0.99)	0.19‐1.00	18.4	56.4	—
NPSLE^i^ discrimination (n=7)	0.88 (0.76‐0.95)	0.54‐0.98	0.89 (0.76‐0.95)	0.50‐0.99	18.0	21.7	—
Histopathology (n=7)	0.94 (0.80‐0.98)	0.28‐1.00	0.98 (0.92‐0.99)	0.60‐1.00	48.2	43.4	—
MRI^j^ (n=8)	0.91 (0.81‐0.96)	0.53‐0.99	0.88 (0.79‐0.94)	0.61‐0.97	24.5	13.9	—
Raman spectroscopy (n=3)	0.97 (0.90‐0.99)	0.83‐0.99	0.97 (0.92‐0.99)	0.88‐0.99	—	—	—
Clinical images (n=5)	0.90 (0.86‐0.93)	0.79‐0.95	0.92 (0.88‐0.95)	0.82‐0.97	—	—	—
External validation (n=9)	0.90 (0.77‐0.96)	0.32‐0.99	0.92 (0.83‐0.96)	0.53‐0.99	38.2	27.0	—

a PI reflects expected diagnostic performance range in a new similar study. NA, not computed for subgroups with <4 studies. Aliyev et al [48] and de Araujo et al [33] were excluded from all analyses. In the all-table exploratory analysis, studies reporting results for multiple algorithms contribute multiple rows (each representing an independent algorithmic comparison, not double-counting of patients)*.*

b PI: 95% prediction interval.

c *I²*: between-study heterogeneity.

d ML: machine learning.

e Not assessed.

f DL: deep learning.

g SLE: systemic lupus erythematosus.

h LN: lupus nephritis.

i NPSLE: neuropsychiatric systemic lupus erythematosus.

j MRI: magnetic resonance imaging.

### Subgroup and Sensitivity Analyses

#### Subgroup Analysis by Validation Strategy

The model performance was significantly different between internal and external validation. Studies using only internal validation (20 studies) [8,16,17,19,20,22,25-27,29-31,34-37,39,41,43,44] showed a pooled sensitivity of 0.92 (95% CI 0.89‐0.94) and specificity of 0.94 (95% CI 0.91‐0.96). Studies using independent external validation (9 studies) [21,23,24,28,32,38,40,42,45] showed a pooled sensitivity of 0.90 (95% CI 0.77‐0.96, 95% PI 0.32‐0.99) and specificity of 0.92 (95% CI 0.83‐0.96, 95% PI 0.53‐0.99). Nonetheless, only 9 [21,23,24,28,32,38,40,42,45] of 29 (31%) studies [8,16,17,19-32,34-45] performed independent external validation.

#### Other Subgroup Analyses

**Figure 7.**
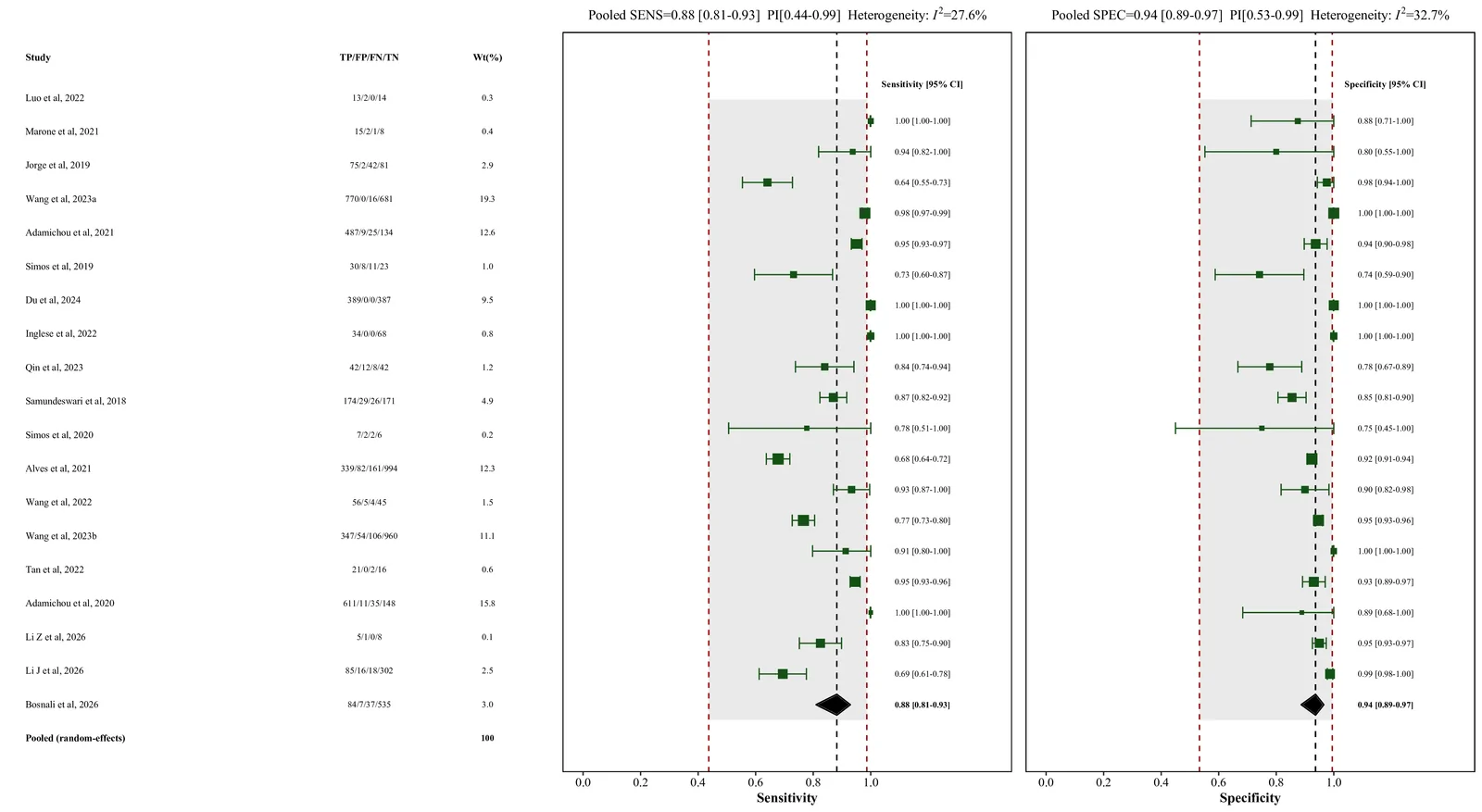
Cochrane-style forest plot for traditional machine learning models (19 studies): sensitivity and specificity [8,17,19,21-29,32,37-39,43-45]. Random-effects bivariate model with Knapp-Hartung adjustment; dashed red lines=95% prediction interval. FN: false negative; FP: false positive; ML: machine learning; PI: prediction interval; SENS: sensitivity; SPEC: specificity; TN: true negative; TP: true positive; Wt: study weight.

**Figure 8.**
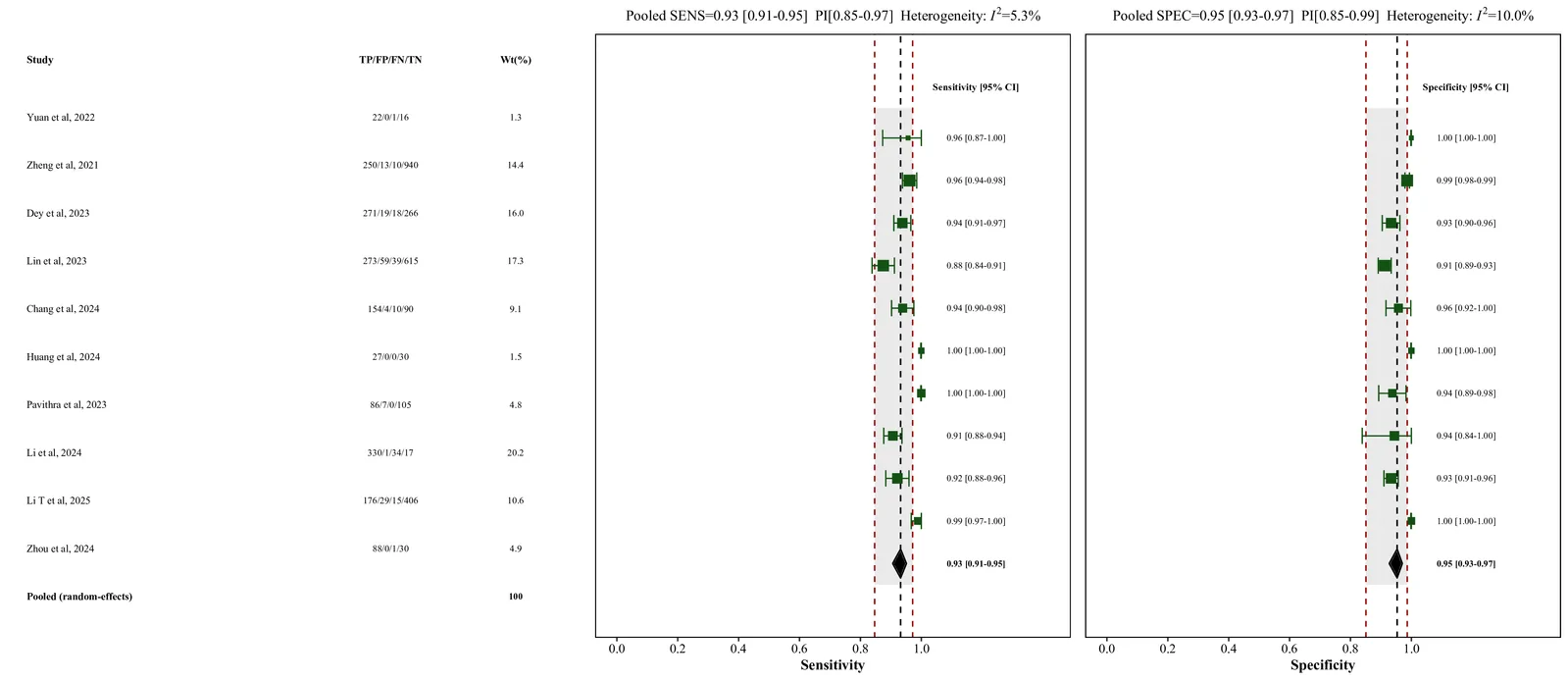
Cochrane-style forest plot for deep learning models (10 studies): sensitivity and specificity [16,20,30,31,34-36,40-42]. Random-effects bivariate model with Knapp-Hartung adjustment; dashed red lines=95% prediction interval. PI: prediction interval; SENS: sensitivity; SPEC: specificity.

**Figure 9.**
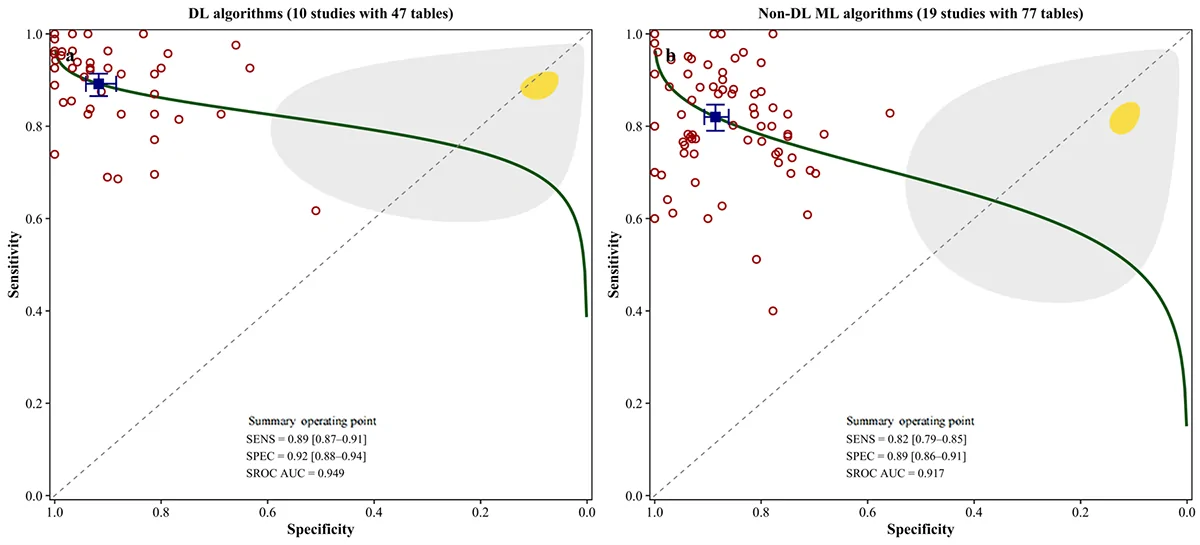
Summary receiver operating characteristic curves stratified by algorithm type. (A) Deep learning algorithms (10 studies with 47 tables) [16,20,30,31,34-36,40-42] and (B) non–deep learning machine learning algorithms (19 studies with 77 tables) [8,17,19,21-29,32,37-39,43-45]. AUC: area under the curve; DL: deep learning; ML: machine learning; SENS: sensitivity; SPEC: specificity; SROC: summary receiver operating characteristic.

In the all-table exploratory analysis, non-DL ML algorithms (19 studies with 77 tables) [8,17,19,21-29,32,37-39,43-45] showed a pooled sensitivity of 0.82 (95% CI 0.79‐0.85) and specificity of 0.89 (95% CI 0.86‐0.91), with an SROC AUC of 0.917. DL algorithms (10 studies with 47 tables) [16,20,30,31,34-36,40-42] showed a pooled sensitivity of 0.89 (95% CI 0.87‐0.91) and specificity of 0.92 (95% CI 0.88‐0.94), with an SROC AUC of 0.949. In the primary analysis (one table per study), DL models (n=10) [16,20,30,31,34-36,40-42] achieved a pooled sensitivity of 0.93 (95% CI 0.91‐0.95, 95% PI 0.85‐0.97) and a specificity of 0.95 (95% CI 0.93‐0.97, 95% PI 0.85‐0.99), compared with traditional ML models (n=19) [8,17,19,21-29,32,37-39,43-45] at sensitivity 0.88 (95% CI 0.81‐0.93, 95% PI 0.44‐0.99) and specificity 0.94 (95% CI 0.89‐0.97, 95% PI 0.53‐0.99). DL models showed lower heterogeneity (*I*²=5.3% for sensitivity, *I*²=10.0% for specificity) than ML models (*I*²=27.6% for sensitivity, *I*²=32.7% for specificity) and narrower PIs, suggesting more consistent performance across studies. The Cochrane-style forest plots for ML and DL subgroups are shown in Figures 7 and 8, and the subgroup ROC comparison by algorithm type is shown in Figure 9. The forest plots for ML and DL algorithm subgroups are also presented in Figure S5 in Multimedia Appendix 3.

Subgroup analysis by data modality revealed that models using histopathology images (7 studies) [20-24,29,32] yielded a pooled sensitivity of 0.94 (95% CI 0.80‐0.98) and specificity of 0.98 (95% CI 0.92‐0.99). MRI-based models (8 studies) [16,17,25,28,36,38,39,45] yielded a pooled sensitivity of 0.91 (95% CI 0.81‐0.96) and specificity of 0.88 (95% CI 0.79‐0.94). Raman spectroscopy–based models (3 studies) [34,35,41] yielded a pooled sensitivity of 0.97 (95% CI 0.90‐0.99) and specificity of 0.97 (95% CI 0.92‐0.99). Clinical image-based models (5 studies) [27,30,40,42,44] yielded a pooled sensitivity of 0.90 (95% CI 0.86‐0.93) and specificity of 0.92 (95% CI 0.88‐0.95). No statistically significant differences in performance across modalities were identified in formal subgroup testing. The pooled SROC performance by data modality is presented in Figure S6 in Multimedia Appendix 3, and the forest plots for different feature types are presented in Figure S7 in Multimedia Appendix 3.

Subgroup analysis by sample size revealed that studies with ≥100 participants had lower pooled sensitivity (0.89 vs 0.93) and specificity (0.92 vs 0.96) than studies with <100 participants, indicating that studies with a small sample size overestimate model performance.

#### Sensitivity Analyses

Leave-one-out analysis showed that no single study had a disproportionate impact on the pooled estimates for any of the 3 primary tasks. Excluding studies with a high overall risk of bias or a small sample size (<50 participants) did not significantly change the pooled sensitivity or specificity for the primary analyses, confirming the robustness of the results.

#### Small-Study Effects Assessment

**Figure 10.**
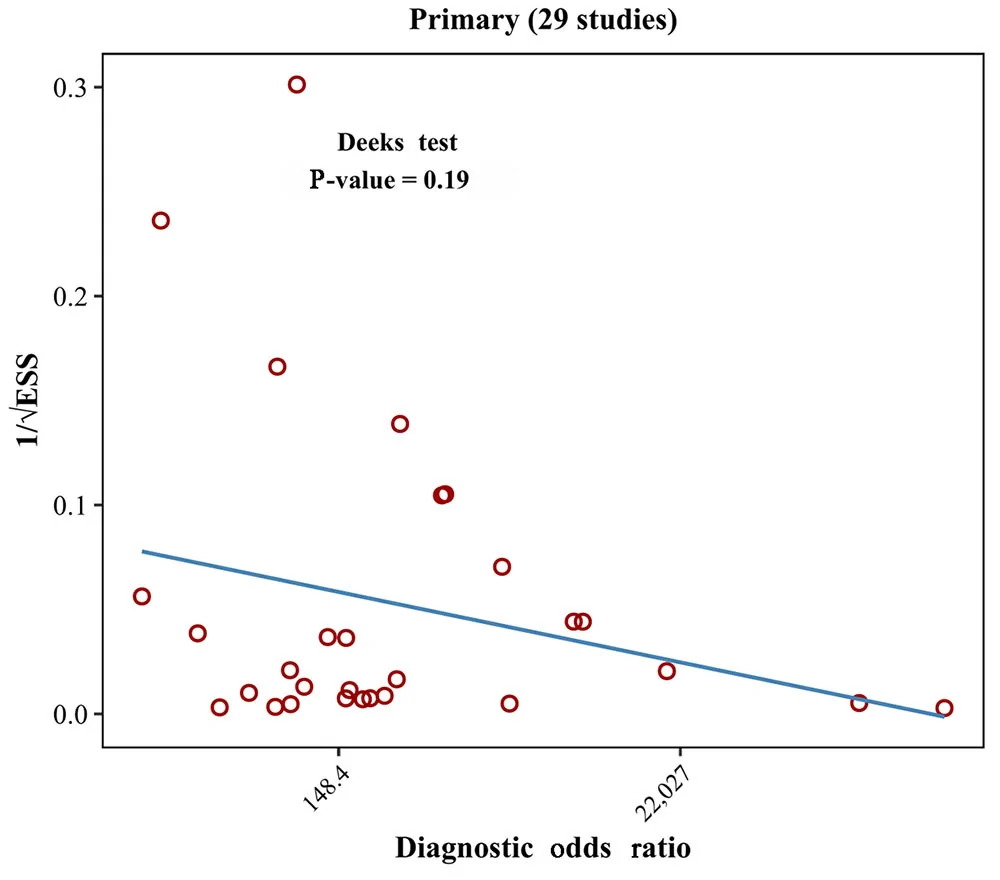
Deeks funnel plot asymmetry test for the primary analysis (29 studies) [8,16,17,19-32,34-45]. *P*=.19, indicating no significant small-study effects. ESS: effective sample size.

Visual inspection of the funnel plot showed no obvious asymmetry, and the Deeks asymmetry test found no statistically significant small-study effects (*P*=.19 for the primary task-stratified analysis; *P*=.16 for the exploratory all-table analysis; Figure 10). However, we explicitly note that this analysis has limited statistical power due to the small number of included studies, and cannot rule out researcher-driven optimism bias from selective reporting of prespecified models, threshold tuning, or post hoc model selection, which is a pervasive limitation in ML diagnostic research. The Deeks funnel plots for exploratory analyses are presented in Figure S9 in Multimedia Appendix 3.

#### Comparison Between ML Models and Human Clinicians

Only 2 studies reported head-to-head comparisons between ML models and human clinicians using the same held-out test dataset, with a total of 6 diagnostic contingency tables. Due to the sparse data, wide CIs, heterogeneous clinical tasks, and varying levels of clinician expertise across studies, no inferential meta-analysis or formal between-group comparison was performed; an exploratory descriptive SROC analysis is presented in Figure S8 in Multimedia Appendix 3 for visualization only. In both studies, ML models showed higher overall sensitivity than clinicians, with comparable specificity, but the small number of studies and heterogeneous methods implied that no definitive conclusions can be drawn about the relative diagnostic performance of ML versus human clinicians.

## Discussion

### Summary of Principal Findings

Our principal finding is that ML models show promising in-sample diagnostic accuracy across all 3 prespecified clinical tasks. In the primary task-stratified analyses, the pooled sensitivity and specificity were 0.90 and 0.94, respectively, for SLE classification (n=17) [8,21,23,24,27,29-31,34-37,40-44]; 0.93 and 0.95, respectively, for LN diagnosis (n=5) [19,20,22,26,32]; and 0.88 and 0.89, respectively, for NPSLE discrimination (n=7) [16,17,25,28,38,39,45], and SROC AUC values of 0.940 (SLE), 0.938 (LN), and 0.877 (NPSLE) in the all-table exploratory analyses. However, these promising results must be interpreted with caution for several reasons. First, all included studies were retrospective, and only 9 [21,23,24,28,32,38,40,42,45] of 29 (31%) [8,16,17,19-32,34-45] studies performed independent external validation. Second, while between-study heterogeneity was substantially lower in the updated analysis than the previous version (*I*²=23.9% overall for sensitivity, compared with *I*²=97.5% previously), the PIs remained wide for most analyses, reflecting significant variations in model performance across different clinical populations, imaging modalities, and institutional contexts [49,50]. Third, reference standards were inconsistent across studies, with some relying on retrospective clinical labels rather than prospectively applied standardized classification criteria [51]. Fourth, no included study assessed model calibration, DCA, or net clinical benefit, suggesting that high AUC values do not prove that the model is ready for clinical application [52]. Fifth, the GRADE assessment confirmed high certainty of evidence for most analyses but low certainty for LN diagnosis due to inconsistency and imprecision. Hence, additional studies are needed before pooled LN estimates can be confidently applied in clinical practice [46,47]. Our analysis also confirms that internal validation systematically overestimates ML model performance relative to external validation, which is commonly observed across diagnostic ML research and is not unique to SLE [53,54].

### Comparison With Existing Literature and Innovations of This Review

Our findings build on and address the fundamental limitations of prior systematic reviews in this field, with 4 key contributions to the literature. First, we addressed the core conceptual flaw of prior reviews by performing fully task-stratified meta-analyses, rather than pooling heterogeneous clinical tasks into a single analysis [55]. This ensures the clinical interpretability and methodological validity of our pooled estimates and aligns with the core assumptions of DTA meta-analysis. Prior reviews have combined SLE classification, LN activity grading, NPSLE discrimination, and disease activity estimation into a single pooled analysis, yielding results that lack practical value in both statistical and clinical terms. Our task-stratified approach resolves this critical issue [56]. Furthermore, this task-stratified approach revealed significant differences in the certainty of evidence across tasks: while SLE classification and NPSLE discrimination achieved high GRADE certainty, LN diagnosis was rated as low certainty, a distinction that would have been obscured by pooling all tasks together.

Second, we use state-of-the-art statistical methods for DTA meta-analysis, including the HKSJ random-effects model and 95% PIs, which can account for extreme between-study heterogeneity [49]. Unlike prior reviews that overinterpreted pooled AUC values in the setting of *I*²>95%, we explicitly acknowledged the limitations of pooled point estimates with extreme heterogeneity and used PIs to quantify the real-world variability in model performance [57]. For instance, the primary analysis for SLE classification showed a relatively narrow CI (sensitivity 0.84‐0.94) but a wide PI (sensitivity 0.53‐0.99). It indicates that while the average performance is precisely estimated, a new study in a different population could yield markedly different results [35]. This avoids the statistical misinterpretations in previous reviews that led to inaccurate conclusions [58].

Third, we implemented strict rules for including contingency tables to eliminate data dependency, double counting, and optimism bias. Prior reviews extracted multiple nonindependent contingency tables from the same study (from multiple models, thresholds, or cross-validation splits), treating them as independent observations and leading to artificially narrow CIs and inflated precision [59]. In our primary analyses, we adhered to the principle of using only one independent table per study and excluded post hoc best-performing model results, thereby resolving this methodological flaw [60]. Fourth, we systematically distinguished between internal and external validation results and quantified the overestimation of performance from internal validation [61,62]. Prior reviews have confounded internal and external validation results, leading to overestimations of real-world generalizability [63]. Internal validation methods, such as k-fold cross-validation and random split-sample validation, estimate model performance within the same dataset used for training, and are therefore susceptible to optimistic bias from dataset-specific patterns, overfitting to demographic or institutional characteristics, and data leakage. In contrast, independent external validation tests the model on entirely new data from different institutions, time periods, or geographic settings, providing a more realistic estimate of real-world performance [54,62]. In the updated analysis, 9 [21,23,24,28,32,38,40,42,45] of 29 (31%) [8,16,17,19-32,34-45] studies performed external validation, and the PI for externally validated studies (sensitivity 0.32‐0.99, specificity 0.53‐0.99) underscores the high variability of ML model performance across clinical contexts. We prioritized external validation results in all primary analyses [64].

### Limitations of the Included Studies

The included studies have several major methodological limitations that severely undermine the validity and clinical applicability of their findings. First, all included studies were retrospective in design, which introduces a high risk of bias, including selection bias, data leakage, and overfitting. Retrospective ML model development typically relies on convenience samples that do not reflect the target clinical population, and models may learn spurious correlations from the data that do not generalize to real-world settings [65]. No prospective studies of ML for SLE diagnosis were identified in this review. Second, the vast majority of studies used only internal validation, with only 31% (9/29) performing independent external validation. Internal validation is well known to systematically overestimate diagnostic accuracy, particularly in ML studies with flexible modeling pipelines and limited sample sizes [53]. Our subgroup analysis confirmed this, showing significantly lower performance in externally validated studies [56]. Without independent, multicenter external validation, the real-world generalizability of these models remains unproven [66].

Third, despite the low overall between-study heterogeneity (*I*²=23.9%), the PIs remained wide for most analyses, indicating that the expected performance of ML models in any single new clinical setting could vary substantially. This is partially explained by differences in data modality, validation strategy, and sample size [50]. The remaining heterogeneity is likely due to differences in study population, reference standard definition, model development workflow, and preprocessing steps, which limit the generalizability of pooled estimates [67]. The wide 95% PIs for most analyses confirm that model performance is highly variable across different clinical settings and populations [54]. Fourth, there was significant inconsistency in the reference standards used across studies. Some studies used retrospective clinical labels rather than standardized classification criteria, and a small number of studies used reference standards that were partially informed by the same data used to train the ML model, thereby introducing the risk of circular validation and bias in the reference standard [51]. This inconsistency further limits the comparability of results across studies [55].

Fifth, researcher-driven optimism bias is a pervasive risk across all included studies. Many studies reported only results of the best-performing model, without prespecifying the primary model or accounting for multiple comparisons [68]. This selective reporting leads to an overestimation of model performance and cannot be ruled out even in the absence of statistically significant small-study effects [69]. Finally, none of the included studies assessed the clinical utility of their models. High AUC values alone do not prove that the model can improve patient outcomes in clinical practice; a model with high discriminative accuracy may still cause net harm if it is poorly calibrated, its decision threshold is inappropriate for the target clinical population, or it leads to overdiagnosis, unnecessary invasive testing, or inappropriate immunosuppressive treatment. Model calibration refers to the agreement between the model’s predicted probabilities and the actual observed outcome frequencies across the probability range, and is typically assessed using calibration plots, the Hosmer-Lemeshow test, or the expected calibration error (ECE). DCA is the recommended framework for evaluating whether a diagnostic model provides net clinical benefit over default strategies (treat-all or treat-none) across a range of clinically plausible decision thresholds [52]. Without such formal assessments and prospective evaluation of the model’s impact on diagnostic workflow, patient management decisions, and clinical outcomes, it is impossible to determine whether these ML models would provide meaningful benefit to patients with SLE in real-world clinical practice.

### Limitations of This Systematic Review

This review has several limitations that should be acknowledged. First, we only included studies published in English, which may introduce language bias [70]. Second, we excluded conference abstracts, which may lead to publication bias, as negative or nonsignificant results are less likely to be published in full-text peer-reviewed journals. Third, the number of included studies for some subgroup analyses was small, limiting statistical power, and these analyses are therefore presented as exploratory only. Fourth, we were unable to perform meta-analysis of individual patient data, which would have allowed for more robust adjustment for confounding factors and more detailed subgroup analyses [71]. Finally, we were unable to assess the risk of data leakage in the included studies, as this is often not reported in detail, which may lead to an overestimation of model performance in the original studies [72,73]. Additionally, the GRADE framework, while widely used for assessing certainty of evidence, has limitations when applied to DTA studies. It was originally developed for intervention studies and may not fully capture all sources of uncertainty in diagnostic accuracy meta-analyses [46].

### Implications for Future Research and Clinical Practice

The findings of this review have critical implications for future research and clinical practice. For future research, we make the following evidence-based recommendations. First, future ML studies for SLE diagnosis must be prospectively designed, with preregistration of the study protocol, model development plan, and statistical analysis plan, to reduce the risk of bias and selective reporting [65,68]. Second, all ML models must undergo independent, multicenter external validation in cohorts that reflect the target clinical population, to confirm real-world generalizability [54,62,66]. Third, future studies must use clearly defined, clinically homogeneous diagnostic tasks and consistent reference standards to reduce heterogeneity and enable meaningful comparison between models [55,69]. Fourth, all future studies must assess clinical utility, including model calibration, DCA to quantify net clinical benefit, and evaluation of the model’s impact on diagnostic workflow and patient outcomes [52]. Fifth, future studies should perform prespecified, head-to-head comparisons between ML models and board-certified rheumatologists, using standardized test datasets and clearly defined clinician expertise levels, to determine the incremental value of ML-assisted diagnosis over standard clinical care.

For clinical practice, our review confirms that ML models for SLE diagnosis are not yet ready for routine clinical application [74]. The current evidence is of variable quality, with GRADE certainty ranging from high (for most analyses) to low (for LN diagnosis), due to exclusively retrospective designs, lack of independent external validation, wide PIs, and absence of clinical utility assessment [75]. There is currently no evidence that these models improve patient outcomes in real-world clinical settings [76]. At this stage, ML should only be used as an auxiliary tool to support expert clinical judgment, in the context of prospective research studies, until robust, externally validated, clinically beneficial models are developed [77,78].

### Conclusion

This is the first methodologically rigorous, task-stratified systematic review and meta-analysis of ML models for SLE diagnosis, with formal GRADE assessment of certainty of evidence, addressing the core conceptual and statistical flaws of prior syntheses. ML models show promising in-sample diagnostic accuracy for 3 distinct SLE-related clinical tasks, but the current evidence is of variable certainty (high for most analyses, low for LN diagnosis), limited by pervasive methodological weaknesses, including exclusively retrospective designs, lack of independent external validation, wide PIs despite low *I*², and absence of clinical utility assessment. The distinction between the narrow CIs (reflecting precision of the average effect) and the wide PIs (reflecting expected variability across new settings) is critical for clinical interpretation: the pooled estimates should not be taken as guaranteed performance in any individual new setting [35]. Future research must prioritize prospectively registered, multicenter, externally validated studies with standardized clinical tasks, harmonized reference standards, and formal assessment of clinical utility, to support the safe and effective translation of ML tools into clinical care for SLE.

## Supplementary material

10.2196/90209 Multimedia Appendix 1 Deviations from PROSPERO preregistered protocol (CRD42024545109).

10.2196/90209 Multimedia Appendix 2 Full database search strategies (Cochrane-Compliant).

10.2196/90209 Multimedia Appendix 3 Study characteristics, model development and validation, diagnostic performance, subgroup analyses, human-clinician comparisons, and publication bias.

10.2196/90209 Multimedia Appendix 4 Grading of Recommendations Assessment, Development, and Evaluation summary-of-findings tables for the diagnostic accuracy of machine learning and deep learning models for systemic lupus erythematosus–related diagnostic tasks.

10.2196/90209 Checklist 1 PRISMA 2020 checklist.

10.2196/90209 Checklist 2 PRISMA-DTA complete compliance checklist.

10.2196/90209 Checklist 3 PRISMA-S complete compliance checklist.

10.2196/90209 Checklist 4 PRISMA 2020 complete compliance checklist.

## Abbreviations

ACR: American College of Rheumatology
AUC: area under the curve
CT: computed tomography
DCA: decision-curve analysis
DL: deep learning
DTA: diagnostic test accuracy
ECE: expected calibration error
EEG: electroencephalogram
EHR: electronic health record
EULAR: European League Against Rheumatism
FN: false negative
FODI: finger optical diffusion imaging
FP: false positive
FTIR: Fourier transform infrared spectroscopy
GRADE: Grading of Recommendations Assessment, Development and Evaluation
HKSJ: Hartung-Knapp-Sidik-Jonkman
HSROC: hierarchical summary receiver operating characteristic
ISN: International Society of Nephrology
LN: lupus nephritis
ML: machine learning
MRI: magnetic resonance imaging
NPSLE: neuropsychiatric systemic lupus erythematosus
NPV: negative predictive value
OCT: optical coherence tomography
PI: prediction interval
PPV: positive predictive value
PRISMA: Preferred Reporting Items for Systematic Reviews and Meta-Analyses
PRISMA-DTA: PRISMA for Diagnostic Test Accuracy
PRISMA-S: Preferred Reporting Items for Systematic Reviews and Meta-Analyses literature search extension
PROSPERO: International Prospective Register of Systematic Reviews
QUADAS-AI: Quality Assessment of Diagnostic Accuracy Studies for Artificial Intelligence
ROC: receiver operating characteristic
RPS: Renal Pathology Society
SLE: systemic lupus erythematosus
SLICC: Systemic Lupus Collaborating Clinics
SROC: summary receiver operating characteristic
TN: true negative
TP: true positive
