# Supplementary material for: Diagnostic Performance of Machine Learning for Systemic Lupus Erythematosus: Systematic Review and Meta-Analysis
Source: J Med Internet Res. 2026 Sep 4;28:e90209. doi: 10.2196/90209 (PMC13544417; doi:10.2196/90209)
Supplement: Supplementary file 1 [file jmir-v28-e90209-s002.docx]

1.PubMed Full Search Strategy

#1 "Systemic Lupus Erythematosus"[MeSH Terms] OR "Lupus Erythematosus, Systemic"[Title/Abstract] OR SLE[Title/Abstract] OR "Lupus Nephritis"[MeSH Terms] OR "Lupus Nephritis"[Title/Abstract] OR "Neuropsychiatric Lupus Erythematosus"[MeSH Terms] OR NPSLE[Title/Abstract]

#2 "Machine Learning"[MeSH Terms] OR "Artificial Intelligence"[MeSH Terms] OR "Deep Learning"[MeSH Terms] OR ML[Title/Abstract] OR DL[Title/Abstract] OR "Neural Networks, Computer"[MeSH Terms] OR "Support Vector Machine"[Title/Abstract] OR "Random Forest"[Title/Abstract] OR "Logistic Regression"[Title/Abstract] OR "Decision Tree"[Title/Abstract] OR "Gradient Boosting"[Title/Abstract] OR "Convolutional Neural Network"[Title/Abstract] OR CNN[Title/Abstract]

#3 "Diagnostic Tests, Routine"[MeSH Terms] OR "Diagnostic Accuracy"[Title/Abstract] OR "Sensitivity and Specificity"[MeSH Terms] OR "ROC Curve"[MeSH Terms] OR "AUC"[Title/Abstract] OR "Area Under the Curve"[Title/Abstract] OR "Diagnosis"[MeSH Terms] OR "Diagnostic"[Title/Abstract]

#4 #1 AND #2 AND #3

#5 Filters: English, Publication date from 2014/01/01 to 2026/04/30, Humans

2. Embase Full Search Strategy

#1 'systemic lupus erythematosus'/exp OR 'systemic lupus erythematosus':ti,ab OR sle:ti,ab OR 'lupus nephritis'/exp OR 'lupus nephritis':ti,ab OR 'neuropsychiatric systemic lupus erythematosus'/exp OR npsle:ti,ab

#2 'machine learning'/exp OR 'machine learning':ti,ab OR 'artificial intelligence'/exp OR 'artificial intelligence':ti,ab OR 'deep learning'/exp OR 'deep learning':ti,ab OR ml:ti,ab OR dl:ti,ab OR 'neural network'/exp OR 'support vector machine':ti,ab OR 'random forest':ti,ab OR 'convolutional neural network':ti,ab OR cnn:ti,ab

#3 'diagnostic test accuracy'/exp OR 'diagnostic accuracy':ti,ab OR 'sensitivity and specificity'/exp OR 'sensitivity':ti,ab OR 'specificity':ti,ab OR 'roc curve'/exp OR 'auc':ti,ab OR 'area under curve':ti,ab OR 'diagnosis'/exp OR 'diagnostic':ti,ab

#4 #1 AND #2 AND #3

#5 [english]/lim AND [2014/01/01-2026/04/30]/lim AND [human]/lim

3. Cochrane Library Full Search Strategy

#1 MeSH descriptor: [Systemic Lupus Erythematosus] explode all trees OR "systemic lupus erythematosus" OR SLE OR "lupus nephritis" OR "neuropsychiatric lupus" OR NPSLE:ti,ab,kw

#2 MeSH descriptor: [Machine Learning] explode all trees OR MeSH descriptor: [Artificial Intelligence] explode all trees OR "machine learning" OR "deep learning" OR ML OR DL OR "neural network" OR "support vector machine" OR "random forest" OR "convolutional neural network" OR CNN:ti,ab,kw

#3 MeSH descriptor: [Diagnostic Tests] explode all trees OR "diagnostic accuracy" OR "sensitivity and specificity" OR "ROC curve" OR AUC OR "area under the curve" OR diagnosis OR diagnostic:ti,ab,kw

#4 #1 AND #2 AND #3

#5 Date: 2014 to 2026; Language: English; Study type: Diagnostic Test Accuracy Studies

4. Web of Science Core Collection Full Search Strategy

TS=("systemic lupus erythematosus" OR SLE OR "lupus nephritis" OR "neuropsychiatric lupus" OR NPSLE)

AND TS=("machine learning" OR "artificial intelligence" OR "deep learning" OR ML OR DL OR "neural network" OR "support vector machine" OR "random forest" OR "convolutional neural network" OR CNN)

AND TS=("diagnostic accuracy" OR "sensitivity and specificity" OR "ROC curve" OR AUC OR "area under the curve" OR diagnosis OR diagnostic)

AND LANGUAGE: (English)

AND Timespan: 2014-01-01 to 2026-04-30

AND Document types: Article

AND Indexes: SCI-EXPANDED, SSCI

5. IEEE Xplore Full Search Strategy

("Document Title": "systemic lupus erythematosus" OR "Document Title": SLE OR "Document Title": "lupus nephritis" OR "Document Title": NPSLE OR "Abstract": "systemic lupus erythematosus" OR "Abstract": SLE OR "Abstract": "lupus nephritis" OR "Abstract": NPSLE)

AND ("Document Title": "machine learning" OR "Document Title": "deep learning" OR "Document Title": "artificial intelligence" OR "Abstract": "machine learning" OR "Abstract": "deep learning" OR "Abstract": "neural network")

AND ("Document Title": "diagnostic accuracy" OR "Document Title": "sensitivity and specificity" OR "Document Title": AUC OR "Abstract": "diagnostic accuracy" OR "Abstract": "sensitivity" OR "Abstract": "specificity")

AND Publication Year: 2014 to 2026

AND Language: English
