# Supplementary material for: Diagnostic Performance of Machine Learning for Systemic Lupus Erythematosus: Systematic Review and Meta-Analysis
Source: J Med Internet Res. 2026 Sep 4;28:e90209. doi: 10.2196/90209 (PMC13544417; doi:10.2196/90209)
Supplement: Supplementary file 2 [file jmir-v28-e90209-s003.docx]

**Supplementary Material 3**

Table S1. Methods of model training and validation (29 studies)

Table S2. Indicators, algorithms and data sources (29 studies)

Table S3. Study design and basic demographics (29 studies)

Table S4. Terminology mapping across included studies (29 studies)

Table S5. Implementation/reporting checklist

Fig. S1 Pooled overall SROC performance (primary and exploratory).

Fig. S2 Forest plot of primary task-stratified analysis (29 studies).

Fig. S3 Forest plot of all-table exploratory analysis (124 tables, 4 parts).

Fig. S4 Forest plot of different disease types (SLE, LN, NPSLE).

Fig. S5 Forest plot of different algorithm types (ML, DL).

Fig. S6 Pooled SROC performance by data modality.

Fig. S7 Forest plot of different feature types.

Fig. S8 Pooled performance comparison with human clinicians.

Fig. S9 Publication bias (Deeks’ funnel plots).

**Table S1. Methods of model training and validation (29 studies)**

| **Author (Year)** | **Reference Standard** | **Internal Validation** | **External Validation** |
| --- | --- | --- | --- |
| Yuan et al. (2022) | Expert consensus | 3-fold CV | No |
| Dey et al. (2023) | Consistent label | NR | No |
| Alves et al. (2021) | Expert consensus | NR | No |
| Adamichou et al. (2021) | Expert consensus | 10-fold CV | Yes |
| Du et al. (2024) | Consistent label | 10-fold CV | Yes |
| Simos et al. (2020) | Consistent label | Random split | Yes |
| Zheng et al. (2021) | Expert consensus | 10-fold CV | No |
| Marone et al. (2021) | Consistent label | LOOCV | No |
| Inglese et al. (2022) | Consistent label | Random split | No |
| Samundeswari et al. (2018) | Histopathology | 10-fold CV | No |
| Wang et al. (2023a) | Consistent label | 10-fold CV | No |
| Luo et al. (2022) | Consistent label | 4-fold CV | No |
| Wang et al. (2023b) | Consistent label | 10-fold CV | No |
| Pavithra et al. (2023) | Consistent label | Random split | No |
| Chang et al. (2024) | Histopathology | 5-fold CV | No |
| Simos et al. (2019) | Expert consensus | 5-fold CV | Yes |
| Lin et al. (2023) | Consistent label | 5-fold CV | No |
| Jorge et al. (2019) | Consistent label | Random split | Yes |
| Qin et al. (2023) | Consistent label | 5-fold CV | No |
| Tan et al. (2022) | Consistent label | k-fold CV | No |
| Li et al. (2024) | Expert consensus | Random split | Yes |
| Huang et al. (2024) | Histopathology | 5-fold CV | No |
| Wang et al. (2022) | Consistent label | 10-fold CV | Yes |
| Adamichou et al. (2020) | Expert consensus | NR | No |
| Zhou et al. (2024) | Histopathology | 5-fold CV | No |
| Bosnali et al. (2026) | Expert consensus | 10-fold CV | No |
| Li T et al. (2025) | Consistent label | Random split | Yes |
| Li Z et al. (2026) | Expert consensus | 5-fold CV | Yes |
| Li J et al. (2026) | Expert consensus | 5-fold CV | No |

*NR, not reported; CV, cross-validation; LOOCV, leave-one-out CV. Aliyev et al. (2025) excluded due to high risk of bias. de Araujo et al. (2023) excluded.*

**Table S2. Indicators, algorithms and data sources (29 studies)**

| **Author (Year)** | **Device** | **Algorithm** | **ML/DL** | **Transfer Learning** | **N (train/test/int/ext)** | **Data Range** | **Open Access** |
| --- | --- | --- | --- | --- | --- | --- | --- |
| Yuan et al. (2022) | MRI | WRFE-A | DL | No | 26/13/NR/NR | NR | No |
| Dey et al. (2023) | Clinical images | CNN | DL | Yes | NR | NR | Yes |
| Alves et al. (2021) | Histopathology | SLEDAI | ML | No | 3504/NR/1576/NR | 2013-2020 | Yes |
| Adamichou et al. (2021) | Histopathology | LASSO-LR | ML | No | 722/80/NR/655 | 2005-2019 | No |
| Du et al. (2024) | Histopathology | RF | ML | No | 776/86/NR/198 | 2008-2016 | No |
| Simos et al. (2020) | MRI | RF | ML | No | 66/17/NR/NR | NR | No |
| Zheng et al. (2021) | Histopathology | CNN | DL | No | 973/37/NR/NR | 2011-2019 | No |
| Marone et al. (2021) | FODI | SVM-L | ML | No | 191/1/NR/NR | 2018-2020 | No |
| Inglese et al. (2022) | MRI | SVM | ML | No | 102/NR/NR/NR | 2007-2015 | No |
| Samundeswari (2018) | Clinical images | MLP | ML | No | 360/40/NR/NR | NR | No |
| Wang et al. (2023a) | Histopathology | XGB | ML | No | 1320/147/NR/NR | 2017-2022 | No |
| Luo et al. (2022) | MRI | Linear SVM | ML | No | 84/28/NR/NR | 2014-2020 | No |
| Wang et al. (2023b) | Histopathology | SLEDAI-2K | ML | No | 1320/147/NR/NR | 2017-2022 | No |
| Pavithra et al. (2023) | MRI | 5LAM | DL | No | 138/30/30/NR | NR | Yes |
| Chang et al. (2024) | Raman | SNN | DL | No | 266/66/NR/NR | NR | No |
| Simos et al. (2019) | MRI | SVM (SVC) | ML | No | 58/14/NR/NR | 2015-2016 | No |
| Lin et al. (2023) | OCT | OCTNet | DL | No | 1512/432/NR/NR | 2018-2022 | No |
| Jorge et al. (2019) | Histopathology | EHR | ML | No | 200/NR/NR/NR | NR | Yes |
| Qin et al. (2023) | Ultrasound | MLP | ML | No | 104/45/NR/NR | 2019-2022 | No |
| Tan et al. (2022) | MRI | SVM | ML | No | 35/4/NR/NR | 1997-1999 | No |
| Li et al. (2024) | Clinical images | MMDLS | DL | No | 310/64/72/NR | 2010-2021 | No |
| Huang et al. (2024) | Raman | DBRAN | DL | No | 46/11/NR/NR | NR | No |
| Wang et al. (2022) | Histopathology | HMFO-SVM | ML | No | 88/22/NR/NR | 2009-2019 | No |
| Adamichou et al. (2020) | Histopathology | NR | ML | No | NR | 2005-2016 | No |
| Zhou et al. (2024) | Raman/IR/Metab | CMACF | DL | No | 286/71/NR/NR | NR | No |
| Bosnali et al. (2026) | EHR/Lab | LWL/Auto-Weka | ML | No | NR | NR | No |
| Li T et al. (2025) | Retinal fundus | ViT (DeepSLE) | DL | Yes | NR/NR/NR/4 cohorts | Multi-center | No |
| Li Z et al. (2026) | MRI | LDA | ML | No | NR/NR/NR/ext | NR | No |
| Li J et al. (2026) | Nailfold VC | XGBoost | ML | No | NR | NR | No |

*NR, not reported; CNN, convolutional neural network; ML, machine learning; DL, deep learning; SVM, support vector machine; RF, random forest; MLP, multilayer perceptron; XGB, XGBoost; OCT, optical coherence tomography; FODI, finger optical diffusion imaging; EHR, electronic health record; LDA, linear discriminant analysis; VC, videocapillaroscopy.*

**Table S3. Study design and basic demographics (29 studies)**

| **Author (Year)** | **Task** | **Design** | **N** | **Mean Age (SD)** | **Female %** | **Positive Cases** | **Country** |
| --- | --- | --- | --- | --- | --- | --- | --- |
| Yuan et al. (2022) | NPSLE | Retro | 39 | 30.8 (NR) | 82.1% | 23 | China |
| Dey et al. (2023) | SLE | Retro | 574 | NR | NR | 289 | NR |
| Alves et al. (2021) | SLE | Retro | 5080 | 50.4 (14.5) | 93.2% | 3504 | USA |
| Adamichou et al. (2021) | SLE | Retro | 802 | NR | NR | 401 | Greece |
| Du et al. (2024) | SLE | Retro | 862 | 41.0 (13.3) | 83.1% | 432 | China |
| Simos et al. (2020) | NPSLE | Retro | 83 | 43.6 (14.0) | 92.8% | 44 | Greece |
| Zheng et al. (2021) | LN | Retro | 349 | NR | 89.4% | 163 | China |
| Marone et al. (2021) | LN | Retro | 26 | 37.3 (10.8) | 92.3% | 16 | USA |
| Inglese et al. (2022) | NPSLE | Retro | 163 | 41.5 (13.0) | 90.2% | 43 | Netherlands |
| Samundeswari (2018) | SLE | Retro | 400 | NR | NR | 200 | India |
| Wang et al. (2023a) | LN | Retro | 1467 | NR | NR | 786 | China |
| Luo et al. (2022) | NPSLE | Retro | 112 | NR | 68.8% | 49 | China |
| Wang et al. (2023b) | SLE | Retro | 1467 | NR | NR | 453 | China |
| Pavithra et al. (2023) | SLE | Retro | 198 | NR | NR | 86 | NR |
| Chang et al. (2024) | SLE | Retro | 332 | NR | NR | 164 | China |
| Simos et al. (2019) | NPSLE | Retro | 72 | 45.3 (13.5) | 83.3% | 41 | Greece |
| Lin et al. (2023) | SLE | Retro | 6476 | 40.4 (19.1) | 76.3% | 2160 | China |
| Jorge et al. (2019) | SLE | Retro | 400 | 54.8 (15.2) | 87.4% | 114 | USA |
| Qin et al. (2023) | LN | Retro | 149 | 37.2 (12.4) | 78.5% | 66 | China |
| Tan et al. (2022) | NPSLE | Retro | 39 | 30.8 (NR) | NR | 23 | China |
| Li et al. (2024) | SLE | Retro | 382 | NR | NR | 364 | China |
| Huang et al. (2024) | SLE | Retro | 57 | NR | NR | 27 | China |
| Wang et al. (2022) | LN | Retro | 110 | NR | NR | 50 MLN; 60 PLN | China |
| Adamichou et al. (2020) | SLE | Retro | 1091 | NR | NR | 690 | Greece |
| Zhou et al. (2024) | SLE | Retro | 357 | NR | NR | 30 SLE; 59 LN | China |
| Bosnali et al. (2026) | SLE | Retro | NR | NR | NR | NR | Turkey |
| Li T et al. (2025) | SLE | Retro | NR | NR | NR | NR | China/UK |
| Li Z et al. (2026) | NPSLE | Retro | NR | NR | NR | NR | China |
| Li J et al. (2026) | SLE | Retro | NR | NR | NR | NR | China |

*NR, not reported; SLE, systemic lupus erythematosus; LN, lupus nephritis; NPSLE, neuropsychiatric SLE; MLN, membranous LN; PLN, proliferative LN; Retro, retrospective.*

**Table S4. Terminology mapping across included studies (29 studies)**

| **Study ID** | **Authors’ term (verbatim)** | **Aligned term** | **Split unit** | **Int/Ext** |
| --- | --- | --- | --- | --- |
| Yuan_2022 | 3-fold cross-validation | Internal validation | Patient | Internal |
| Dey_2023 | NR | Internal validation | Patient | Internal |
| Alves_2021 | NR | Internal validation | Patient | Internal |
| Adamichou_2021 | 10-fold cross validation | External validation | Patient | External |
| Du_2024 | 10-fold cross-validation | External validation | Patient | External |
| Simos_2020 | Random split-sample | External validation | Patient | External |
| Zheng_2021 | 10-fold cross-validation | Internal validation | Patient | Internal |
| Marone_2021 | leave-one-out CV | Internal validation | Patient | Internal |
| Inglese_2022 | Random split-sample | Internal validation | Patient | Internal |
| Samundeswari_2018 | 10-fold cross validation | Internal validation | Patient | Internal |
| Wang_2023a | 10-fold cross validation | Internal validation | Patient | Internal |
| Luo_2022 | 4-fold cross validation | Internal validation | Patient | Internal |
| Wang_2023b | tenfold crossvalidation | Internal validation | Patient | Internal |
| Pavithra_2023 | Random split-sample | Internal validation | Patient | Internal |
| Chang_2024 | five-fold cross-validation | Internal validation | Patient | Internal |
| Simos_2019 | five-fold cross-validation | External validation | Patient | External |
| Lin_2023 | five-fold cross-validation | Internal validation | Patient | Internal |
| Jorge_2019 | Random split-sample | External validation | Patient | External |
| Qin_2023 | five-fold cross validation | Internal validation | Patient | Internal |
| Tan_2022 | k-fold cross-validation | Internal validation | Patient | Internal |
| Li_2024 | Random split-sample | External validation | Patient | External |
| Huang_2024 | five-fold cross-validation | Internal validation | Patient | Internal |
| Wang_2022 | 10-fold cross validation | External validation | Patient | External |
| Adamichou_2020 | NR | Internal validation | Patient | Internal |
| Zhou_2024 | 5-fold cross validation | Internal validation | Patient | Internal |
| Bosnali_2026 | 10-fold cross-validation | Internal validation | Patient | Internal |
| Li_T_2025 | Random split-sample | External validation | Patient | External |
| Li_Z_2026 | Random split-sample | External validation | Patient | External |
| Li_J_2026 | 5-fold cross-validation | Internal validation | Patient | Internal |

*NR, not reported.*

**Table S5. Implementation/reporting checklist**

| **Item** | **What to report** | **Where to report** | **Minimum expectation** |
| --- | --- | --- | --- |
| Data harmonization | Scanner/vendor, protocol alignment, preprocessing | Methods | Site/vendor listed; preprocessing reproducible |
| Splitting strategy | Patient-level train/val/test; leakage checks | Methods & Box 1 | No patient overlap; leakage ruled out |
| External testing | Site-held external set details | Methods/Results | Clear site/time split; n reported |
| Calibration | Method & threshold selection | Methods/Results | Reliability plot/ECE; clinical threshold rationale |
| Decision-curve | Net benefit vs thresholds | Results | Curve + interpretation |
| Error analysis | Clinician-adjudicated error review | Results/Discussion | Representative cases; failure modes |
| Monitoring | Drift & subgroup fairness plan | Discussion | Metrics, triggers, escalation path |

*ECE, expected calibration error.*

**Supplementary Figures**

Fig. S1 Pooled overall SROC performance of ML algorithms.
(a) Primary analysis (29 studies with 29 tables). SENS = 0.91, SPEC = 0.94, AUC = 0.969.

(b) Exploratory all-table analysis (29 studies with 124 tables). SENS = 0.85, SPEC = 0.90, AUC = 0.931.

Fig. S2 Forest plot of primary task-stratified analysis (29 studies, 1 table per study). I² sensitivity = 23.9%, I² specificity = 22.9%. Green squares = study effect size, dashed red = 95% PI.

**Fig. S3 Forest plot of all-table exploratory analysis (124 tables from 29 studies).**

(a) Part 1/4: Traditional ML rows 1–39.

(b) Part 2/4: Traditional ML rows 40–77.

(c) Part 3/4: Deep Learning rows 1–24.

(d) Part 4/4: Deep Learning rows 25–47. Overall: I² sens = 15.2%, I² spec = 22.8%.

**Fig. S4 Forest plot of different disease types.**

(a) SLE classification (n=17). SENS = 0.90 (0.84–0.94), SPEC = 0.94 (0.92–0.96). I² sens = 24.7%, I² spec =6.7%.

(b) LN diagnosis (n=5). SENS = 0.93 (0.86–0.97), SPEC = 0.95 (0.76–0.99). I² sens = 18.4%, I² spec = 56.4%.

(c) NPSLE discrimination (n=7). SENS = 0.88 (0.76–0.95), SPEC = 0.89 (0.76–0.95). I² sens = 18.0%, I² spec = 21.7%.

**Fig. S5 Forest plot of different algorithm types.**

(a) Traditional ML (n=19). SENS = 0.88 (0.81–0.93), SPEC = 0.94 (0.89–0.97). I² sens = 27.6%, I² spec = 32.7%.

(b) Deep learning (n=10). SENS = 0.93 (0.91–0.95), SPEC = 0.95 (0.93–0.97). I² sens = 5.3%, I² spec = 10.0%.

Fig. S6 Pooled SROC performance by data modality. (a) Histopathology (n=7): AUC = 0.985; (b) MRI (n=8): AUC = 0.952; (c) Raman spectroscopy (n=3): AUC = 0.987; (d) Clinical images (n=5): AUC = 0.960.

**Fig. S7 Forest plot of different feature types.**

(a) Histopathology (n=7). SENS = 0.94 (0.80–0.98), SPEC = 0.98 (0.92–0.99). I² sens = 48.2%, I² spec = 43.4%.

(b) Raman spectroscopy (n=3). SENS = 0.97 (0.90–0.99), SPEC = 0.97 (0.92–0.99).

(c) MRI (n=8). SENS = 0.91 (0.81–0.96), SPEC = 0.88 (0.79–0.94). I² sens = 24.5%, I² spec = 13.9%.

(d) Clinical images (n=5). SENS = 0.90 (0.86–0.93), SPEC = 0.92 (0.88–0.95).

Fig. S8 Pooled performance comparison of ML/DL with human clinicians (2 studies with 6 tables). SENS = 0.82 (0.71–0.89), SPEC = 0.90 (0.88–0.91), AUC = 0.922.

**Fig. S9 Publication bias (Deeks’ funnel plots).**

Exploratory all-table analysis (124 tables). Deeks’ p = 0.163.
