# Supplementary material for: Diagnostic Performance of Machine Learning for Systemic Lupus Erythematosus: Systematic Review and Meta-Analysis
Source: J Med Internet Res. 2026 Sep 4;28:e90209. doi: 10.2196/90209 (PMC13544417; doi:10.2196/90209)
Supplement: Supplementary file 3 [file jmir-v28-e90209-s004.docx]

# **Table 1. GRADE Summary of Findings: Diagnostic Test Accuracy of Machine Learning/Deep Learning Models for Systemic Lupus Erythematosus (SLE)**

*Population: Patients suspected of systemic lupus erythematosus (SLE), lupus nephritis (LN), or neuropsychiatric SLE (NPSLE). Index test: machine learning/deep learning models. Reference standard: 2019 EULAR/ACR classification criteria, renal biopsy (ISN/RPS classification), 1999 ACR NPSLE case definitions, or expert consensus, as applicable to each outcome.*

**Outcome 1: *Machine learning/deep learning models for the diagnosis of SLE in patients suspected of systemic lupus erythematosus (Overall, 29 studies)***

| **Sensitivity** | 0.91 (95% CI: 0.86 to 0.94) |
| --- | --- |
| **Specificity** | 0.94 (95% CI: 0.91 to 0.96) |
| **Prevalence (assumed)** | 50% |

| **Outcome** | **No. of studies (No. of patients)** | **Study design** | **Factors that may decrease certainty of evidence** | | | | | **Effect per 1,000 patients tested** | **Test accuracy CoE** |
| --- | --- | --- | --- | --- | --- | --- | --- | --- | --- |
|  |  |  | **Risk of bias** | **Indirectness** | **Inconsistency** | **Imprecision** | **Publication bias** |  |  |
| **True positives (patients with the target condition)** | 29 studies | Cross-sectional (cohort type accuracy study) | not serious | not serious | not serious | not serious | none | 455 (430 to 470) | **●●●● High** |
| **False negatives (patients incorrectly classified as not having the disease)** |  |  |  |  |  |  |  | 45 (30 to 70) |  |
| **True negatives (patients without the target condition)** | 29 studies | Cross-sectional (cohort type accuracy study) | not serious | not serious | not serious | not serious | none | 470 (455 to 480) | **●●●● High** |
| **False positives (patients incorrectly classified as having the disease)** |  |  |  |  |  |  |  | 30 (20 to 45) |  |

**Outcome 2: *Traditional machine learning models for the diagnosis of SLE in patients suspected of systemic lupus erythematosus (19 studies)***

| **Sensitivity** | 0.88 (95% CI: 0.81 to 0.93) |
| --- | --- |
| **Specificity** | 0.94 (95% CI: 0.89 to 0.97) |
| **Prevalence (assumed)** | 50% |

| **Outcome** | **No. of studies (No. of patients)** | **Study design** | **Factors that may decrease certainty of evidence** | | | | | **Effect per 1,000 patients tested** | **Test accuracy CoE** |
| --- | --- | --- | --- | --- | --- | --- | --- | --- | --- |
|  |  |  | **Risk of bias** | **Indirectness** | **Inconsistency** | **Imprecision** | **Publication bias** |  |  |
| **True positives (patients with the target condition)** | 19 studies | Cross-sectional (cohort type accuracy study) | not serious | not serious | not serious | not serious | none | 440 (405 to 465) | **●●●● High** |
| **False negatives (patients incorrectly classified as not having the disease)** |  |  |  |  |  |  |  | 60 (35 to 95) |  |
| **True negatives (patients without the target condition)** | 19 studies | Cross-sectional (cohort type accuracy study) | not serious | not serious | not serious | not serious | none | 470 (445 to 485) | **●●●● High** |
| **False positives (patients incorrectly classified as having the disease)** |  |  |  |  |  |  |  | 30 (15 to 55) |  |

**Outcome 3: *Deep learning models for the diagnosis of SLE in patients suspected of systemic lupus erythematosus (10 studies)***

| **Sensitivity** | 0.93 (95% CI: 0.91 to 0.95) |
| --- | --- |
| **Specificity** | 0.95 (95% CI: 0.93 to 0.97) |
| **Prevalence (assumed)** | 50% |

| **Outcome** | **No. of studies (No. of patients)** | **Study design** | **Factors that may decrease certainty of evidence** | | | | | **Effect per 1,000 patients tested** | **Test accuracy CoE** |
| --- | --- | --- | --- | --- | --- | --- | --- | --- | --- |
|  |  |  | **Risk of bias** | **Indirectness** | **Inconsistency** | **Imprecision** | **Publication bias** |  |  |
| **True positives (patients with the target condition)** | 10 studies | Cross-sectional (cohort type accuracy study) | not serious | not serious | not serious | not serious | none | 465 (455 to 475) | **●●●● High** |
| **False negatives (patients incorrectly classified as not having the disease)** |  |  |  |  |  |  |  | 35 (25 to 45) |  |
| **True negatives (patients without the target condition)** | 10 studies | Cross-sectional (cohort type accuracy study) | not serious | not serious | not serious | not serious | none | 475 (465 to 485) | **●●●● High** |
| **False positives (patients incorrectly classified as having the disease)** |  |  |  |  |  |  |  | 25 (15 to 35) |  |

**Outcome 4: *Machine learning/deep learning models for the diagnosis of SLE classification in patients suspected of systemic lupus erythematosus (17 studies)***

| **Sensitivity** | 0.90 (95% CI: 0.84 to 0.94) |
| --- | --- |
| **Specificity** | 0.94 (95% CI: 0.92 to 0.96) |
| **Prevalence (assumed)** | 50% |

| **Outcome** | **No. of studies (No. of patients)** | **Study design** | **Factors that may decrease certainty of evidence** | | | | | **Effect per 1,000 patients tested** | **Test accuracy CoE** |
| --- | --- | --- | --- | --- | --- | --- | --- | --- | --- |
|  |  |  | **Risk of bias** | **Indirectness** | **Inconsistency** | **Imprecision** | **Publication bias** |  |  |
| **True positives (patients with the target condition)** | 17 studies | Cross-sectional (cohort type accuracy study) | not serious | not serious | not serious | not serious | none | 450 (420 to 470) | **●●●● High** |
| **False negatives (patients incorrectly classified as not having the disease)** |  |  |  |  |  |  |  | 50 (30 to 80) |  |
| **True negatives (patients without the target condition)** | 17 studies | Cross-sectional (cohort type accuracy study) | not serious | not serious | not serious | not serious | none | 470 (460 to 480) | **●●●● High** |
| **False positives (patients incorrectly classified as having the disease)** |  |  |  |  |  |  |  | 30 (20 to 40) |  |

**Outcome 5: *Machine learning/deep learning models for the diagnosis of lupus nephritis in patients suspected of lupus nephritis (5 studies)***

| **Sensitivity** | 0.93 (95% CI: 0.86 to 0.97) |
| --- | --- |
| **Specificity** | 0.95 (95% CI: 0.76 to 0.99) |
| **Prevalence (assumed)** | 50% |

| **Outcome** | **No. of studies (No. of patients)** | **Study design** | **Factors that may decrease certainty of evidence** | | | | | **Effect per 1,000 patients tested** | **Test accuracy CoE** |
| --- | --- | --- | --- | --- | --- | --- | --- | --- | --- |
|  |  |  | **Risk of bias** | **Indirectness** | **Inconsistency** | **Imprecision** | **Publication bias** |  |  |
| **True positives (patients with the target condition)** | 5 studies | Cross-sectional (cohort type accuracy study) | not serious | not serious | serious ¹ | serious ² | none | 465 (430 to 485) | **●●○○ Low** |
| **False negatives (patients incorrectly classified as not having the disease)** |  |  |  |  |  |  |  | 35 (15 to 70) |  |
| **True negatives (patients without the target condition)** | 5 studies | Cross-sectional (cohort type accuracy study) | not serious | not serious | serious ¹ | serious ² | none | 475 (380 to 495) | **●●○○ Low** |
| **False positives (patients incorrectly classified as having the disease)** |  |  |  |  |  |  |  | 25 (5 to 120) |  |

**Outcome 6: *Machine learning/deep learning models for the discrimination of NPSLE in patients suspected of neuropsychiatric SLE (7 studies)***

| **Sensitivity** | 0.88 (95% CI: 0.76 to 0.95) |
| --- | --- |
| **Specificity** | 0.89 (95% CI: 0.76 to 0.95) |
| **Prevalence (assumed)** | 50% |

| **Outcome** | **No. of studies (No. of patients)** | **Study design** | **Factors that may decrease certainty of evidence** | | | | | **Effect per 1,000 patients tested** | **Test accuracy CoE** |
| --- | --- | --- | --- | --- | --- | --- | --- | --- | --- |
|  |  |  | **Risk of bias** | **Indirectness** | **Inconsistency** | **Imprecision** | **Publication bias** |  |  |
| **True positives (patients with the target condition)** | 7 studies | Cross-sectional (cohort type accuracy study) | not serious | not serious | not serious | not serious | none | 440 (380 to 475) | **●●●● High** |
| **False negatives (patients incorrectly classified as not having the disease)** |  |  |  |  |  |  |  | 60 (25 to 120) |  |
| **True negatives (patients without the target condition)** | 7 studies | Cross-sectional (cohort type accuracy study) | not serious | not serious | not serious | not serious | none | 445 (380 to 475) | **●●●● High** |
| **False positives (patients incorrectly classified as having the disease)** |  |  |  |  |  |  |  | 55 (25 to 120) |  |

**Outcome 7: *Machine learning/deep learning models for the diagnosis of SLE, LN, or NPSLE in externally validated cohorts (9 studies)***

| **Sensitivity** | 0.90 (95% CI: 0.76 to 0.96) |
| --- | --- |
| **Specificity** | 0.92 (95% CI: 0.83 to 0.96) |
| **Prevalence (assumed)** | 50% |

| **Outcome** | **No. of studies (No. of patients)** | **Study design** | **Factors that may decrease certainty of evidence** | | | | | **Effect per 1,000 patients tested** | **Test accuracy CoE** |
| --- | --- | --- | --- | --- | --- | --- | --- | --- | --- |
|  |  |  | **Risk of bias** | **Indirectness** | **Inconsistency** | **Imprecision** | **Publication bias** |  |  |
| **True positives (patients with the target condition)** | 9 studies | Cross-sectional (cohort type accuracy study) | not serious | not serious | not serious | not serious | none | 450 (380 to 480) | **●●●● High** |
| **False negatives (patients incorrectly classified as not having the disease)** |  |  |  |  |  |  |  | 50 (20 to 120) |  |
| **True negatives (patients without the target condition)** | 9 studies | Cross-sectional (cohort type accuracy study) | not serious | not serious | not serious | not serious | none | 460 (415 to 480) | **●●●● High** |
| **False positives (patients incorrectly classified as having the disease)** |  |  |  |  |  |  |  | 40 (20 to 85) |  |

*CI, confidence interval; CoE, certainty of evidence; NPSLE, neuropsychiatric SLE; LN, lupus nephritis.*

*GRADE Working Group grades of evidence: High certainty (⨁⨁⨁⨁) — we are very confident that the true effect lies close to that of the estimate; Low certainty (⨁⨁◯◯) — our confidence in the effect estimate is limited.*

¹ Rated down for inconsistency: substantial heterogeneity in specificity across studies (I² = 56.4%), exceeding the 50% threshold for downgrading.

² Rated down for imprecision: small number of studies (k = 5) and wide 95% confidence interval for specificity (width = 0.23), exceeding the pre-specified 0.20 threshold.

Risk of bias was assessed using QUADAS-AI; certainty was not rated down for risk of bias, indirectness, or publication bias for any outcome except as noted above. Publication bias was assessed using Deeks' test for the primary and exploratory analyses (p = 0.192 and p = 0.163, respectively; not assessed for individual subgroup analyses due to insufficient studies).
