# Supplementary material for: Diagnostic Performance of Machine Learning for Systemic Lupus Erythematosus: Systematic Review and Meta-Analysis
Source: J Med Internet Res. 2026 Sep 4;28:e90209. doi: 10.2196/90209 (PMC13544417; doi:10.2196/90209)
Supplement: Supplementary file 5 [file jmir-v28-e90209-s006.docx]

**PRISMA-DTA Complete Compliance Checklist**

| **Item No.** | **Reporting Requirement** | **Manuscript Location** | **Compliance Status & Notes** |
| --- | --- | --- | --- |
| 1 | Title identifies the report as a systematic review and meta-analysis of diagnostic test accuracy studies | Manuscript Title | Fully compliant. |
| 2 | Structured abstract includes core DTA elements: target population, index test, reference standard, study design, key results, and conclusions | Full Abstract | Fully compliant. All core DTA elements are included in the abstract. |
| 3 | Introduction describes the clinical context, target population, and decision-making role of the index test | Introduction, Paragraph 1 | Fully compliant. Clinical context and decision-making role of the 3 diagnostic tasks are clearly defined. |
| 4 | Explicitly defines the index test and reference standard, including thresholds and validation requirements | Methods: Study Selection Criteria | Fully compliant. Index test (ML/DL models) and task-specific reference standards are clearly defined. |
| 5 | Specifies inclusion and exclusion criteria for the target population and clinical setting | Methods: Study Selection Criteria | Fully compliant. Target population criteria are clearly defined for each diagnostic task. |
| 6 | Full, reproducible search strategy aligned with PRISMA-S | Methods: Literature Search Strategy; Attachment 2 | Fully compliant. |
| 7 | Describes study selection and data extraction processes, including DTA-specific items | Methods: Study Selection; Data Extraction | Fully compliant. Data extraction form includes all DTA-specific core items. |
| 8 | Specifies risk of bias assessment tool validated for DTA and AI studies | Methods: Risk of Bias Assessment | Fully compliant. QUADAS-AI tool (validated for AI-DTA research) is used. |
| 9 | Specifies meta-analysis methods, including HSROC model and rationale for random-effects model selection | Methods: Statistical Analysis | Fully compliant. Rationale for HKSJ random-effects model and HSROC analysis is provided. |
| 10 | Defines methods for assessing and exploring between-study heterogeneity | Methods: Statistical Analysis; Subgroup and Sensitivity Analyses | Fully compliant. I² and 95% prediction intervals are used to quantify heterogeneity, with subgroup analyses to explore sources. |
| 11 | Reports study characteristics including core DTA elements: target population, index test, reference standard, threshold, and validation strategy | Results: Study Characteristics; Attachment 4: Tables S1-S2 | Fully compliant. |
| 12 | Reports risk of bias results across all domains for included studies | Results: Risk of Bias Assessment; Supplementary Material 4: Fig. S11, Fig. S12 | Fully compliant. |
| 13 | Reports primary diagnostic accuracy results, including pooled sensitivity, specificity, AUC, 95% CIs, and 95% PIs | Results: Primary Task-Stratified Meta-Analyses | Fully compliant. Results are stratified by 3 independent clinical tasks, with no pooling of heterogeneous tasks. |
| 14 | Reports heterogeneity assessment and subgroup analysis results | Results: Primary Analyses; Subgroup and Sensitivity Analyses | Fully compliant. |
| 15 | Discussion addresses DTA-specific limitations, including reference standard variability, spectrum bias, and validation differences | Discussion: Limitations of Included Studies | Fully compliant. All core DTA limitations are discussed in detail. |
| 16 | Discussion addresses clinical applicability of the index test in real-world settings | Discussion: Implications for Clinical Practice; Conclusions | Fully compliant. Explicit statement on the lack of clinical readiness of the models is provided. |
