# Supplementary material for: Diagnostic Performance of Machine Learning for Systemic Lupus Erythematosus: Systematic Review and Meta-Analysis
Source: J Med Internet Res. 2026 Sep 4;28:e90209. doi: 10.2196/90209 (PMC13544417; doi:10.2196/90209)
Supplement: Supplementary file 6 [file jmir-v28-e90209-s007.docx]

**PRISMA-S Complete Compliance Checklist**

| **Item No.** | **Reporting Requirement** | **Manuscript Location** | **Compliance Status & Notes** |
| --- | --- | --- | --- |
| 1 | Reports the lead and expert personnel involved in developing the search (e.g., medical librarian) | Methods: Literature Search Strategy; Attachment 2 | Fully compliant. Explicitly states the search strategy was developed in collaboration with and peer-reviewed by a medical librarian. |
| 2 | Reports all databases and platforms searched, date ranges, and date of last search for each database | Methods: Literature Search Strategy; Attachment 2 | Fully compliant. 5 databases are listed, with full date ranges and last search dates. |
| 3 | Provides the full, unedited search strategy for each database, including all terms, operators, field limits, and filters | Attachment 2 | Fully compliant. Full search strings for all 5 databases are provided. |
| 4 | Reports use of any automation or machine learning tools for screening/searching | Methods: Literature Search Strategy | Fully compliant. Explicitly states no automated tools were used for search or screening. |
| 5 | Reports supplementary search methods, including reference list screening, citation chasing, and grey literature searching | Methods: Literature Search Strategy | Fully compliant. Manual reference list screening is described, with rationale for excluding grey literature provided. |
| 6 | Reports methods for de-duplication of search results | Methods: Study Selection | Fully compliant. EndNote X9 de-duplication with manual cross-checking is described. |
| 7 | Reports peer review of the search strategy (aligned with PRESS guidelines) | Attachment 2 | Fully compliant. Search strategy was reviewed by a medical librarian against the PRESS checklist. |
