# Supplementary material for: Diagnostic Performance of Machine Learning for Systemic Lupus Erythematosus: Systematic Review and Meta-Analysis
Source: J Med Internet Res. 2026 Sep 4;28:e90209. doi: 10.2196/90209 (PMC13544417; doi:10.2196/90209)
Supplement: Supplementary file 7 [file jmir-v28-e90209-s008.docx]

**PRISMA 2020 Complete Compliance Checklist**

| **Section** | **Item No.** | **Reporting Requirement** | **Manuscript Location** | **Compliance Status & Notes** |
| --- | --- | --- | --- | --- |
| Title | 1 | Identify the report as a systematic review with meta-analysis | Manuscript Title | Fully compliant. Title explicitly states "systematic review and meta-analysis". |
| Abstract | 2 | Adhere to PRISMA 2020 structured abstract guidelines | Full Abstract | Fully compliant. Abstract follows the standardized PRISMA 2020 structure: Background, Objectives, Data Sources, Study Eligibility, Study Appraisal and Synthesis Methods, Results, Limitations, Conclusions, and Registration. |
| Introduction | 3 | Describe the rationale for the review in the context of existing evidence | Introduction, Paragraphs 1-3 | Fully compliant. Rationale is grounded in the clinical need for improved SLE diagnostics, limitations of prior ML research, and critical methodological flaws of existing systematic reviews. |
| Introduction | 4 | State explicit research objectives and pre-specified subgroup analyses | Introduction, Paragraph 4; Methods: Subgroup and Sensitivity Analyses | Fully compliant. Primary objectives for 3 independent clinical tasks are clearly stated, with 5 pre-specified subgroup analyses defined a priori. |
| Methods | 5 | Report PROSPERO registration details, including registration number, platform, and access to the protocol | Methods: Protocol and Registration; End of Abstract | Fully compliant. PROSPERO registration number CRD42024545109 is provided, with full deviations from the protocol reported in Attachment 3. |
| Methods | 6 | Specify explicit inclusion and exclusion criteria, including study design, participants, index test, reference standard, outcomes, and setting | Methods: Study Selection Criteria | Fully compliant. Detailed inclusion/exclusion criteria are provided for the 3 pre-specified diagnostic tasks, aligned with DTA review standards. |
| Methods | 7 | Detail all information sources, full search strategies, and date of last search for each database, aligned with PRISMA-S | Methods: Literature Search Strategy; Attachment 2 | Fully compliant. Full search strategies for 5 databases are provided, reviewed by a medical librarian, with clear date ranges and search terms. |
| Methods | 8 | Describe the study selection process, including screening steps, number of reviewers, and conflict resolution | Methods: Study Selection Criteria | Fully compliant. Dual independent screening by two reviewers is described, with conflicts resolved by consensus with a third senior reviewer. |
| Methods | 9 | Describe the data extraction process, data collection form, data verification, and conflict resolution | Methods: Data Extraction | Fully compliant. Triple independent data extraction with cross-validation is described, using a pre-piloted form aligned with the Cochrane Handbook for DTA reviews. |
| Methods | 10 | Specify the risk of bias assessment tool, assessment process, and rating criteria | Methods: Risk of Bias Assessment | Fully compliant. QUADAS-AI tool (gold standard for AI-DTA studies) was used, with dual independent assessment and clear rating criteria. |
| Methods | 11 | Specify the effect measure synthesis methods, including meta-analysis model, effect metrics, and heterogeneity quantification | Methods: Statistical Analysis | Fully compliant. Hartung-Knapp-Sidik-Jonkman (HKSJ) random-effects model is specified, with sensitivity, specificity, AUC, 95% CIs, 95% prediction intervals, and I² statistic reported. |
| Methods | 12 | Describe pre-specified subgroup and sensitivity analyses to explore heterogeneity and validate result robustness | Methods: Subgroup and Sensitivity Analyses | Fully compliant. 5 pre-specified subgroup analyses and 3 sensitivity analyses are defined, with clear rationale. |
| Methods | 13 | Describe methods used to assess small-study effects | Methods: Small-Study Effects Assessment | Fully compliant. Funnel plot and Egger’s test are specified, with explicit note that these assess small-study effects only, not publication bias. |
| Results | 14 | Report study selection flow, including number of records screened, excluded, and included at each stage, with reasons for exclusion | Results: Study Selection; Attachment 6: Figure S1 | Fully compliant. Full selection flow is reported, with a PRISMA 2020-compliant flowchart provided. |
| Results | 15 | Report baseline characteristics of included studies, including study design, sample size, setting, participant demographics, index test, and reference standard | Results: Study Characteristics; Attachment 4: Tables S1-S2 | Fully compliant. Detailed characteristics are reported, stratified by the 3 independent clinical tasks. |
| Results | 16 | Report risk of bias assessments for each included study, across all domains | Results: Risk of Bias Assessment; Attachment 4: Fig. S11, Fig. S12 | Fully compliant. Full QUADAS-AI ratings are reported, with summary and per-study plots provided. |
| Results | 17 | Report main meta-analysis results, including individual study effect sizes, pooled estimates, 95% CIs/PIs, and heterogeneity statistics | Results: Primary Task-Stratified Meta-Analyses; Attachment 4: Fig. S2-S4, Fig. S7, Fig. S9 | Fully compliant. Results are stratified by 3 independent clinical tasks, with full reporting of prediction intervals and heterogeneity, and explicit statements on the limited clinical interpretability of pooled estimates with extreme heterogeneity. |
| Results | 18 | Report results of subgroup and sensitivity analyses, including sources of heterogeneity and robustness of results | Results: Subgroup and Sensitivity Analyses | Fully compliant. Pre-specified subgroup analyses are reported, confirming significant overestimation of performance with internal validation. |
| Results | 19 | Report results of small-study effects assessment | Results: Small-Study Effects Assessment | Fully compliant. Funnel plot and Egger’s test results are reported, with appropriate caveats on interpretation. |
| Discussion | 20 | Summarize principal findings, aligned with study objectives, without redundant numerical results | Discussion: Summary of Principal Findings | Fully compliant. Key findings are summarized concisely, aligned with the review objectives, with no redundant reporting of numerical results. |
| Discussion | 21 | Discuss limitations of the included studies and the review itself | Discussion: Limitations of Included Studies; Limitations of This Systematic Review | Fully compliant. Comprehensive discussion of methodological limitations of the primary research and the review is provided. |
| Discussion | 22 | Discuss findings in the context of existing evidence, including innovations and contributions of the review | Discussion: Comparison with Existing Literature and Innovations of This Review | Fully compliant. Critical comparison with prior systematic reviews is provided, with explicit description of the 4 core innovations of this work. |
| Discussion | 23 | Discuss clinical implications of the findings, including recommendations for future research and clinical practice | Discussion: Implications for Future Research and Clinical Practice; Conclusions | Fully compliant. Evidence-based recommendations for research and clinical practice are provided, with explicit statement that models are not ready for routine clinical use. |
| Other | 24 | Provide Funding Statement, Competing Interests, Author Contributions, Generative AI Use Disclosure, and Data Availability Statement | Statements and Declarations | Fully compliant. All mandatory declarations are provided in standalone sections, aligned with journal requirements. |
| Other | 25 | Provide access to all supplementary materials | Full Attachment Section | Fully compliant. All supplementary materials are provided in full, with clear labeling. |
